# The ASC-1 Complex Disassembles Collided Ribosomes

**DOI:** 10.1016/j.molcel.2020.06.006

**Published:** 2020-08-20

**Authors:** Szymon Juszkiewicz, Shaun H. Speldewinde, Li Wan, Jesper Q. Svejstrup, Ramanujan S. Hegde

**Affiliations:** 1 MRC Laboratory of Molecular Biology, Francis Crick Avenue, Cambridge, CB2 0QH, UK; 2 The Francis Crick Institute, 1 Midland Road, London NW1 1AT, UK

**Keywords:** ribosome, translation, quality control, helicase, ASCC3, RQC

## Abstract

Translating ribosomes that slow excessively incur collisions with trailing ribosomes. Persistent collisions are detected by ZNF598, a ubiquitin ligase that ubiquitinates sites on the ribosomal 40S subunit to initiate pathways of mRNA and protein quality control. The collided ribosome complex must be disassembled to initiate downstream quality control, but the mechanistic basis of disassembly is unclear. Here, we reconstitute the disassembly of a collided polysome in a mammalian cell-free system. The widely conserved ASC-1 complex (ASCC) containing the ASCC3 helicase disassembles the leading ribosome in an ATP-dependent reaction. Disassembly, but not ribosome association, requires 40S ubiquitination by ZNF598, but not GTP-dependent factors, including the Pelo-Hbs1L ribosome rescue complex. Trailing ribosomes can elongate once the roadblock has been removed and only become targets if they subsequently stall and incur collisions. These findings define the specific role of ASCC during ribosome-associated quality control and identify the molecular target of its activity.

## Highlights

• ASC-1 Complex (ASCC) disassembles collided ribosomes ubiquitinated by ZNF598
• ATPase activity of the ASCC3 helicase is needed to split the leading stalled ribosome
• ASCC generates 60S-nascent chains targeted by the ribosome quality control complex
• Dispatch of the lead ribosome by ASCC allows trailing ribosomes to resume translation

## Graphical Abstract

**Figure:**
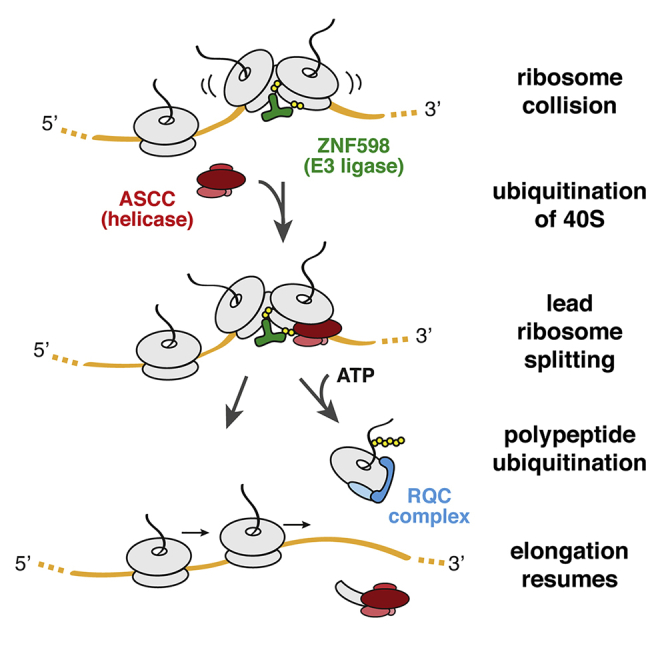

## Teaser

Ribosomes translate mRNAs at ~4 to 6 codons per second. Various mRNA defects cause a ribosome to slow, leading to collisions with trailing ribosomes. Juszkiewicz et al. show that the ASC-1 complex containing the helicase ASCC3 selectively dissociates the lead ribosome of a collision, allowing trailing ribosomes to continue translation.

## Introduction

The rate of elongation during translation of mRNA by a ribosome is non-uniform. Numerous causes of site-specific ribosome slowing have been described. Some slowdowns are beneficial and act to improve dynamic processes, such as co-translational protein folding (Stein et al., 2019), protein targeting to an organelle (Pechmann et al., 2014), and mRNA localization (Yanagitani et al., 2011). Other slowdowns, such as those triggered by a damaged mRNA, are pathological (Doma and Parker, 2006, Joazeiro, 2017).

The degree of ribosome slowing is thought to be a key parameter that distinguishes physiologic pauses from pathologic stalls. Prolonged stalls incur collisions with trailing ribosomes. Several recent studies have found that ribosome collisions are used by the cell as a proxy for aberrant translation to initiate downstream quality control (Ikeuchi et al., 2019, Juszkiewicz et al., 2018, Simms et al., 2017). Thus, considerable attention has now turned to understanding how cells detect collisions, how detection is converted into multiple downstream responses, and how the collided ribosome complex is ultimately resolved.

The distinct 40S-40S interface that characterizes collided ribosomes facilitates their direct recognition by the collision-specific ubiquitin ligase ZNF598 (Juszkiewicz et al., 2018). The homologous protein Hel2 in yeast is thought to serve the same role (Ikeuchi et al., 2019). Ubiquitination of 40S protein targets by ZNF598 or Hel2 is required to abort translation (Garzia et al., 2017, Juszkiewicz and Hegde, 2017, Matsuo et al., 2017, Sundaramoorthy et al., 2017). When ubiquitination is prevented, collided ribosomes continue elongating through an otherwise stall-inducing poly(A) sequence (Juszkiewicz and Hegde, 2017), probably at a slower rate (Chandrasekaran et al., 2019) and with reduced reading frame fidelity (Juszkiewicz and Hegde, 2017, Koutmou et al., 2015). Aborting translation at pathologic collisions is therefore important to minimize the production of aberrant proteins.

How 40S ubiquitination aborts translation is not well understood. The crucial ubiquitination sites on eS10 in mammals (Garzia et al., 2017, Juszkiewicz and Hegde, 2017, Juszkiewicz et al., 2018, Sundaramoorthy et al., 2017) and uS10 in yeast (Matsuo et al., 2017) are far from the sites of elongation factor binding, mRNA decoding, or other key translation reactions. Furthermore, both the yeast and mammalian ubiquitination sites are on flexible tails of the respective target proteins. Thus, a flexible ubiquitin mark on the solvent face of the 40S subunit is somehow converted into a commitment to stop elongation.

Affinity purification of Hel2-engaged ribosomes identified the ribosome quality control trigger (RQT) complex containing the predicted RNA helicase Slh1 (also called Rqt2), Cue3 (also called Rqt3), and Ykr023w (renamed Rqt4) (Matsuo et al., 2017). All three factors were independently identified using genetic screens for ribosome readthrough (Sitron et al., 2017). Cells lacking these factors phenocopy ΔHel2 cells, resulting in a readthrough of model stalling sequences (Matsuo et al., 2017, Sitron et al., 2017) despite elevated accumulation of ribosomes near the stall region (D’Orazio et al., 2019, Ikeuchi et al., 2019, Matsuo et al., 2017, Sitron et al., 2017). Hel2 and Slh1 are therefore needed to abort translation and avoid readthrough at sites of ribosome slowing. Three of four subunits of the ASC-1 complex (ASCC) are homologous to the yeast RQT complex and seem to show similar phenotypes when disrupted in cultured cells (Hashimoto et al., 2020, Matsuo et al., 2017).

Although 40S ubiquitination is required to abort translation, it is not the commitment step because elongation can proceed if downstream factors (e.g., Slh1) are absent (Matsuo et al., 2017, Sitron et al., 2017). Whether additional factors act downstream of the helicase complex is unclear. Two irreversible events that are candidate commitment steps have been described: endonucleolytic cleavage of the mRNA and ribosome subunit dissociation. In yeast, the cleavage reaction (Doma and Parker, 2006, Passos et al., 2009) is dependent on ribosome collisions (Simms et al., 2017) and Cue2 (D’Orazio et al., 2019). Recent studies suggest that endonucleolytic cleavage is ordinarily a minor reaction that is enhanced when cells lack Slh1 (D’Orazio et al., 2019). Thus, Slh1 minimizes the accumulation of collided ribosomes that are otherwise substrates for Cue2-mediated mRNA cleavage.

The other potential commitment step, subunit dissociation of stalled ribosomes, can be mediated by Pelo (Dom34 in yeast), Hbs1L (Hbs1 in yeast), and ABCE1 (Rli1 in yeast) (Pisareva et al., 2011, Shoemaker et al., 2010, Tsuboi et al., 2012). The Pelo-Hbs1L-GTP ternary complex engages the ribosome and Pelo accommodates into the A-site after Hbs1L hydrolyzes GTP and dissociates (Hilal et al., 2016, Pisareva et al., 2011, Shao et al., 2016, Shoemaker et al., 2010). Accommodated Pelo recruits ABCE1, which splits the ribosome into subunits (Becker et al., 2012, Pisareva et al., 2011, Shao et al., 2016, Shoemaker and Green, 2011). The liberated 60S-peptidyl-tRNA complex recruits ribosome-associated quality control (RQC) factors to mediate nascent protein ubiquitination (Lyumkis et al., 2014, Shao et al., 2013, Shao et al., 2015, Shen et al., 2015).

The Pelo-Hbs1-ABCE1 reaction operates most efficiently on ribosomes lacking mRNA in the A-site (Pisareva et al., 2011, Shoemaker et al., 2010), presumably because Pelo partially occupies the mRNA path (Becker et al., 2011, Hilal et al., 2016, Shao et al., 2016). In reconstituted splitting reactions, the mRNA can apparently be displaced locally (Shao and Hegde, 2014, Shao et al., 2016), indicating that an empty A-site is not a strict requirement. Because Dom34 is implicated in various stall-dependent quality control processes in yeast (Bhattacharya et al., 2010, Doma and Parker, 2006, Izawa et al., 2012, Passos et al., 2009), it is possible that the RQT complex facilitates splitting factor activity to abort translation. In mammals, ASCC subunits, Pelo, and Hbs1L were found in a genetic modifier screen of a stall-inducing small molecule (Liaud et al., 2019), but it was not clear whether they act in parallel pathways or as part of a single pathway as proposed by the authors. To begin addressing these issues, we investigated the role of ASCC using a combination of cell-based and biochemical approaches. We find that ASCC acts on ubiquitinated collided ribosomes to selectively disassemble the lead ribosome of a queue by a different pathway than Pelo/Hbs1L-mediated splitting of ribosomes stalled on truncated mRNAs.

## Results

### ASCC Is Required for Aborting Translation at a Ribosome Stall

**Figure 1:**
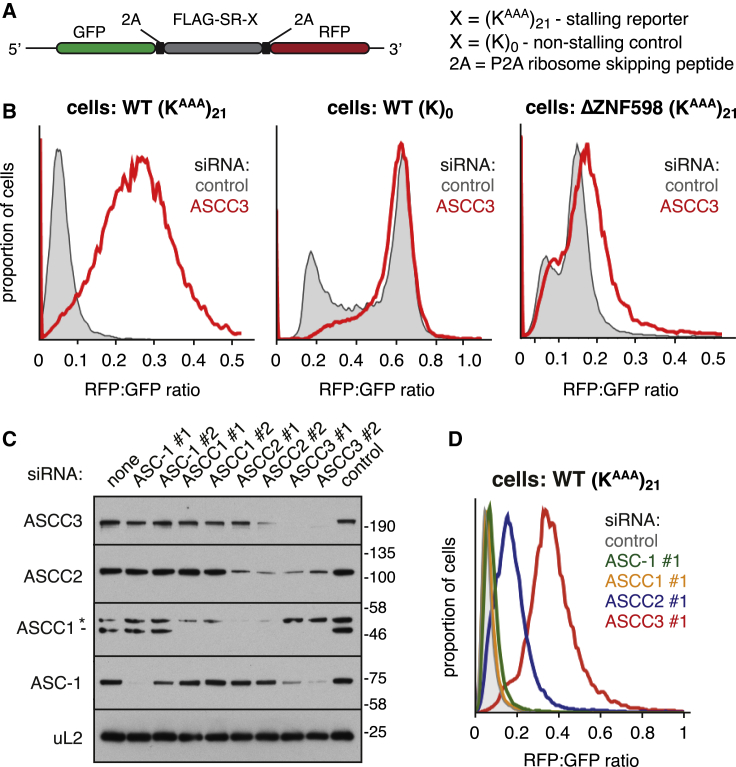
ASCC Is Required for Aborting Translation at a Ribosome Stall (A) The dual-fluorescence reporter used to study translational stalling. (B) Cells were treated with control (gray shaded) or ASCC3-targeting (red traces) siRNAs for 72 h before induction of the stably integrated reporter with doxycycline for 20 h. The RFP:GFP ratio as determined by flow cytometry is plotted as a histogram. (C) Immunoblots of whole cell lysates after treatment with the indicated siRNAs for 72 h. A non-specific product detected by the ASCC1 antibody is indicated with an asterisk in this and subsequent figures. (D) Stalling assay as in (B) after treatment with the indicated siRNAs for 72 h. See also Figures S1 and S5.

Mammalian ASCC3 is an ATP-dependent helicase homologous to yeast Slh1 (Figure S1A). Similar to Slh1 knockout in yeast (Matsuo et al., 2017), knockdown of ASCC3 in cultured mammalian cells allowed increased readthrough of (K^AAA^)_21_, a stalling sequence encoded by 21 AAA Lysine codons (Figures 1A and 1B). In this assay, readthrough is assessed by the ratio of RFP to GFP located after and before the stall (Juszkiewicz and Hegde, 2017). No effect on the RFP:GFP ratio was seen for a (K)_0_ control reporter lacking the stalling sequence (Figure 1B, middle graph) and the effect was almost completely lost in cells knocked out for ZNF598 (Figure 1B, right graph). Thus, like ZNF598 (Garzia et al., 2017, Juszkiewicz and Hegde, 2017, Sundaramoorthy et al., 2017), ASCC3 is needed to terminally abort translation at a poly(A)-mediated stall similar to other stalls (Hashimoto et al., 2020).

ASCC3 is part of the ASC-1 complex with ASC-1 (also known as TRIP4), ASCC1, and ASCC2 (Jung et al., 2002). Sequence homology and domain architecture suggested that ASCC2 is related to yeast Cue3 and ASC-1 is related to Rqt4 (Figure S1A), whereas ASCC1 does not have an obvious homolog in yeast. Depletion of ASCC3 resulted in reduced levels of all other components, supporting their existence in a complex (Figure 1C). ASCC2 knockdown destabilized ASCC1, had a small effect on ASCC3, and had no effect on ASC-1. Knockdown of ASCC1 or ASC-1 did not have any effect on other subunits of the complex. In assays for readthrough of the poly(A) stalling sequence, we observed a partial phenotype for ASCC2 knockdowns and no effect when ASCC1 or ASC-1 were depleted (Figures 1D and S1B).

**Figure 2:**
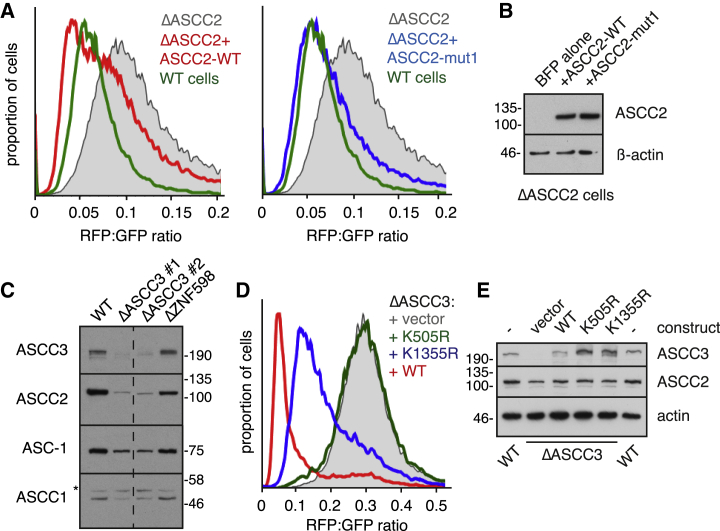
Consequence of ASCC Mutants on Terminal Stalling of Translation (A) ΔASCC2 cells with the stably integrated K(^AAA^)_21_ reporter were transfected with BFP alone (gray trace) or co-transfected with wild-type (WT) ASCC2 (red trace) or CUE-domain mutant ASCC2 (blue trace). After 24 h, the reporter was induced with doxycycline for 20 h before flow cytometry analysis of the transfected cells (identified by BFP expression). Parental WT cells expressing the same reporter at the same locus were analyzed in parallel (green trace). All four sets of cells were analyzed together but separated into two plots for clarity. (B) Immunoblotting for ASCC2 from cells in (A). (C) Two independent knockout clones of ASCC3 were analyzed for the expression of ASCC subunits alongside WT and ΔZNF598 cells. (D) ΔASCC3 cells with the stably integrated K(^AAA^)_21_ reporter were transfected with empty vector (gray trace), WT ASCC3 (red trace), or two different mutant ASCC3 constructs (blue and green traces) and analyzed by flow cytometry. (E) Immunoblots of total cell lysates from the cells in (D). See also Figure S5.

The partial readthrough phenotype of ASCC2 knockdown could be recapitulated in ΔASCC2 knockout cells produced by CRISPR-Cas9 and rescued by re-expression of wild-type ASCC2 (Figures 2A and 2B). Like Cue3 in yeast, ASCC2 contains a ubiquitin-binding CUE domain validated in earlier studies to selectively bind K63-linked ubiquitin with a K_d_ of ~10 μM (Brickner et al., 2017). ASCC2 containing three point mutations that completely abolish the CUE-ubiquitin interaction (Brickner et al., 2017) fully rescued the readthrough phenotype (Figures 2A and 2B). This contrasts with previous suggestions that ubiquitin interaction by Cue3 (Matsuo et al., 2017) and ASCC2 (Hashimoto et al., 2020) is functionally important, a discrepancy that is considered in the Discussion.

Two independent ΔASCC3 clones produced by CRISPR-Cas9 showed undetectable ASCC3 protein and decreased levels of ASCC2, ASCC1 and ASC-1 as expected from the small interfering RNA (siRNA) experiments (Figure 2C). The readthrough phenotype in ΔASCC3 cells mirrored that seen in acute knockdown experiments (Figure 2D, compare to Figure 1B) and could be completely rescued by re-expression of wild-type ASCC3. An ATPase-disrupting point mutation (K505R) in the N-terminal helicase domain showed no rescue of the readthrough phenotype, whereas the analogous mutation of the C-terminal helicase module (K1355R) partially suppressed readthrough (Figure 2D). The wild-type and mutant proteins in these rescue experiments were expressed at comparable levels and close to that seen in wild-type cells (Figure 2E). Thus, ASCC3 is required for preventing readthrough of poly(A), and its loss phenocopies the loss of ZNF598. This activity of ASCC3 is dependent on its ATP-dependent helicase activities and partially on ASCC2, but not ASCC1 or ASC-1.

### The Ribosome Queue at a Site of Ribosome Stalling Is Relieved by ASCC

The requirement of both ZNF598 and ASCC to prevent poly(A) readthrough suggests that they act together to permanently abort translation at sites of stalling. This idea is supported by the observation in yeast that immunoprecipitation of Hel2-containing ribosomes recovers Slh1 (Matsuo et al., 2017). However, the relative roles of ZNF598 and ASCC, their order of action, and their respective effects on ribosome complexes all remain unclear. Given that ZNF598 targets collided ribosomes, and not simply stalled ribosomes, we hypothesized that collisions were also the target for ASCC. To explore this idea, we analyzed the effects of ZNF598 and ASCC when ribosome collisions were induced in a cell-free system.

**Figure 3:**
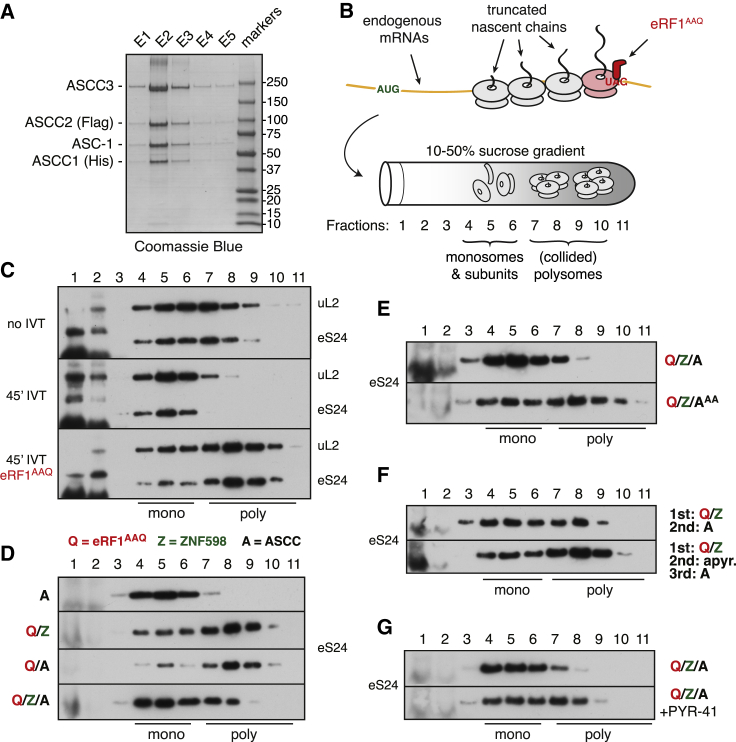
The Ribosome Queue at a Site of Ribosome Stalling Is Relieved by ASCC (A) Elution fractions of purified recombinant human ASCC from insect cells. (B) Schematic of collided polysome production and analysis. (C) Sucrose gradient fractions from rabbit reticulocyte lysate (RRL) analyzed directly (no IVT) or after 45 min of *in vitro* translation (IVT) without or with 0.8 μM eRF1^AAQ^. The migration of ribosomes was detected using anti-eS24 (small subunit) and anti-uL2 (large subunit). The background products seen in fractions 1 and 2 in many blots is due to a large amount of hemoglobin from RRL. (D and E) 45-min translation reactions containing the indicated recombinant proteins were analyzed by sucrose gradient fractionation and immunoblotting for eS24. The following proteins were used: 0.8 μM eRF1^AAQ^, 75 nM ZNF598, and 50 nM ASCC or 50 nM ASCC^AA^ lacking helicase activity. (F) 45-min translation reactions containing 0.8 μM eRF1^AAQ^ and 75 nM ZNF598 were subsequently incubated for 30 min without or with 12.5 U/mL apyrase (to deplete ATP), then supplemented with 50 nM ASCC for another 30 min before sucrose gradient analysis. (G) 15-min translation reactions containing 0.8 μM eRF1^AAQ^ without or with 330 μM E1 inhibitor PYR-41 were supplemented with ZNF598 and ASCC and continued for another 30 min followed by sucrose gradient analysis. See also Figure S2.

The four subunits of ASCC were co-expressed in insect cells and found to purify as a complex in roughly a 1:1:1:1 stoichiometry (Figure 3A). Rabbit reticulocyte lysate (RRL) translation extracts served as the source of translating polysomes. Reticulocytes are naturally devoid of ZNF598 (Juszkiewicz et al., 2018) and ASCC (Figure S2), providing a facile system in which to test their roles in the disassembly of collided ribosomes. We globally induced ribosome collisions by translation elongation of endogenous polysomes in the presence of exogenously added eRF1^AAQ^, a mutant termination factor that competes with endogenous eRF1 to stall ribosomes at the stop codon (Brown et al., 2015, Juszkiewicz et al., 2018). The fate of collided ribosome queues at the stop codon was monitored by immunoblotting for ribosomal proteins after size fractionation by sucrose gradient centrifugation (Figure 3B).

In the absence of eRF1^AAQ^, the ribosomes elongate and terminate normally, resulting in their migration as monosomes and subunits in fractions 4–6 (Figure 3C). Appreciable re-initiation is prevented in this experimental system due to rapid eIF2 phosphorylation by heme-regulated kinase (Trachsel et al., 1978), allowing us to essentially analyze a single-turnover event. In later experiments, this was completely ensured by including the initiation inhibitor pactamycin. In the presence of 0.8 μM eRF1^AAQ^, ~70% of all ribosomes accumulate as polysomes in fractions 7–10 (Figure 3C); these polysomes are collided as judged by electron microscopy and resistance to nuclease digestion (Juszkiewicz et al., 2018). The addition of 75 nM ZNF598 alone or 50 nM ASCC alone had no effect on polysome accumulation induced by eRF1^AAQ^ (Figure 3D). By contrast, addition of ZNF598 with ASCC resulted in mostly monosomes at the expense of polysomes, despite the presence of eRF1^AAQ^. Helicase-deficient mutant ASCC containing the K505A and K1355A mutations (termed ASCC^AA^) had little effect on eRF1^AAQ^-generated polysomes (Figure 3E).

Experiments allowing ribosome queue formation prior to addition of ASCC showed that ASCC acts to relieve the ribosome queue rather than preventing its formation by eRF1^AAQ^ (Figure 3F). In this sequential reaction format, depletion of ATP by apyrase prior to ASCC addition reduced the polysome-to-monosome shift, consistent with ASCC^AA^ being ineffective. Finally, inclusion of an inhibitor of the E1 ubiquitin-activating enzyme during queue formation also stabilized polysomes despite the presence of both ZNF598 and ASCC (Figure 3G). Considered together, these observations demonstrate that ASCC is required to relieve the ribosome queue that forms behind a ribosome stalled at the termination codon. This effect relies on active ubiquitination, indicating that ASCC requires ZNF598 activity, not simply ZNF598 presence. Because ASCC is not needed for ZNF598 engagement and ubiquitination of collided ribosomes (Juszkiewicz et al., 2018), ASCC is likely to act downstream of ZNF598. In all the subsequent analyses focusing on ASCC, ZNF598 was always included to ensure ubiquitination of the collided ribosomes.

### ASCC Acts on Ribosome Queues to Facilitate Subunit Separation

**Figure 4:**
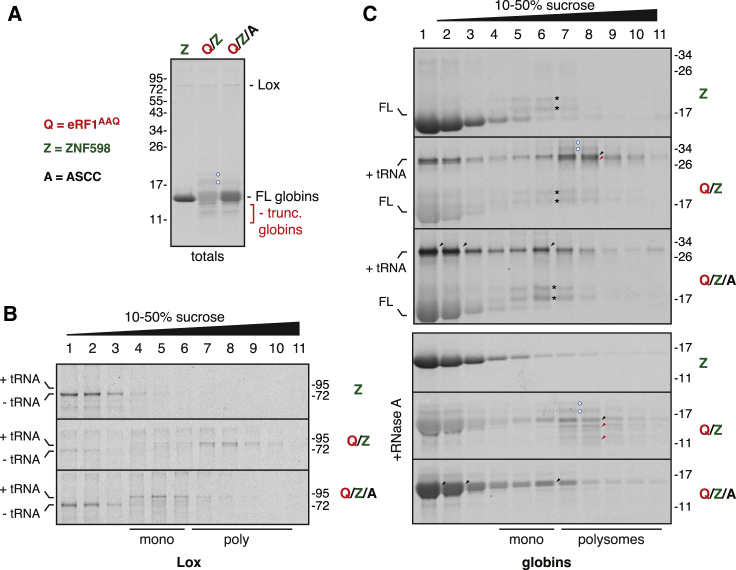
ASCC Acts on Ribosome Queues to Facilitate Subunit Separation (A) *In vitro* translation of endogenous mRNAs in RRL containing ^35^S-methionine, 75 nM ZNF598, 0.8 μM eRF1^AAQ^, and 50 nM ASCC, as indicated. Pactamycin was added after 10 min to inhibit additional initiation. Autoradiogram of the total reaction products, after digestion of tRNA with RNase, is shown. The positions of ~75 kDa lipoxygenase (Lox), ~15 kDa full-length (FL) globins, and truncated globins are indicated. The identity of two ^35^S-labeled products (blue circles) is not known. They are specific to eRF1^AAQ^-containing samples, co-migrate with polysomes (see C), partially diminished with ASCC, and too small to be ubiquitination. (B and C) Reactions as in (A) were separated on sucrose gradients and analyzed under conditions where peptidyl-tRNA products are preserved. The region of the gel showing Lox is displayed in (B), and the region showing globins in the top part of (C). The positions of tRNA-attached polypeptides (verified by their shift upon RNase digestion), and full-length (FL) proteins are indicated. The bottom part of (C) shows the same samples analyzed after digestion with RNase A. The black and red arrows indicate full-length and truncated species of globins, respectively. Black asterisks indicate ^35^S-methionyl-tRNA arising from translation-initiation complexes stabilized by pactamycin. Separate experiments (not shown) verified that this product is only seen with pactamycin and disappears with RNase digestion prior to electrophoresis. A minor product seen between the tRNA-attached and free Lox is probably the product of another endogenous mRNA.

To investigate how ASCC might be relieving a collided ribosome queue, we tracked the ribosome’s nascent polypeptides during the reaction. This was possible because of the exceptionally simple transcriptome of RRL, consisting primarily of mRNAs coding for the ~75 kD lipoxygenase and ~16 kD α- and β-globin. Hence, elongation of endogenous polysomes in the presence of ^35^S-methionine produces two radiolabelled bands corresponding to lipoxygenase and globins (Figure 4A, lane 1). Inclusion of eRF1^AAQ^ should result in incomplete nascent polypeptides of ribosomes queued behind the termination-arrested lead ribosome (see diagram, Figure 3B). These truncated species, observed at the expense of full-length product, can be readily resolved for globins and presumably also exist for lipoxygenase (Figure 4A, lane 2). Inclusion of ASCC increased the ratio of full-length to truncated products (Figure 4A, lane 3). An increase in the full-length product suggests that one consequence of ASCC function is to allow trailing ribosomes to elongate to the stop codon. We come back to this point in the next section.

Separation of these reaction products on a sucrose gradient verified that lipoxygenase normally terminates efficiently, is found in fractions 1–3, and does not contain covalently linked tRNA (Figure 4B). By contrast, ~70%–80% of lipoxygenase is polysome-associated (fractions 7–10) and tRNA-linked in the reaction with eRF1^AAQ^. The ~20%–30% at the top of the gradient presumably represents polypeptides that were terminated by endogenous eRF1 and hence do not have an attached tRNA. Inclusion of ASCC almost completely shifted tRNA-linked lipoxygenase from the polysome fractions to fractions 4–6, containing monosomes and free ribosomal subunits (Figure 4B). The remainder of lipoxygenase is seen in fractions 1–3, lacks tRNA, and has presumably terminated.

Parallel analysis of globin nascent chains was consistent with this conclusion and revealed additional features of ASCC-mediated polysome disassembly. Full-length tRNA-attached nascent chain stalled at the stop codon is seen in the polysome fractions of reactions stalled with eRF1^AAQ^ (Figure 4C). As with lipoxygenase, this species is reduced in the polysome fractions in the presence of ASCC and is increased in the monosome and subunit fractions. Strikingly however, tRNA-attached full-length globins were also increased at the top of the gradient (Figure 4C, fractions 1–3). This population represents peptidyl-tRNA dropoff that accompanies separation of ribosomal subunits. It is not observed for lipoxygenase (and is not completely efficient for globins) because long or folded polypeptides cannot backslide through the 60S exit tunnel (Shao et al., 2013). Thus, ASCC-mediated polysome disassembly ultimately results in ribosome subunit separation leading to either peptidyl-tRNA dropoff or a 60S-peptidyl-tRNA species.

60S-peptidyl-tRNAs are substrates for RQC factors that trigger nascent polypeptide ubiquitination and degradation (Shao et al., 2013, Shao et al., 2015). The reason we do not see nascent chain ubiquitination or a homogeneous 60S population downstream of ASCC in our *in vitro* system is two-fold. First, the RQC factors are at least 100-fold lower in abundance than ribosomes, so extremely little ubiquitination could be realistically expected under conditions when the majority of ribosomes in the lysate are routed into this pathway. Second, 60S-peptidyl-tRNA re-associates readily with 40S unless prevented by inter-subunit binding factors, such as RQC (Shao et al., 2013). Thus, the separated subunit intermediates are not long-lived. Nevertheless, the existence of globin-tRNA drop-off products allows us to infer that subunit separation must have occurred. Thus, in our *in vitro* system, ASCC is a key factor that links ribosome collisions to RQC via polysome disassembly.

### The Lead Ribosome of a Queue Is Targeted by ASCC

As noted above, polysomes stalled by eRF1^AAQ^ contain full-length peptidyl-tRNA within the lead ribosome (Figure 4C, black arrows) and truncated peptidyl-tRNA species in the trailing ribosomes (red arrows). These products are visible in tRNA-containing samples (Figure 4C, top gels) but are particularly well-resolved if the tRNA is removed by RNase prior to electrophoresis (Figure 4C, bottom gels). We noticed that in ASCC-containing samples, these truncated products were substantially diminished in the polysome fraction but did not appear in either the free or monosome fractions. Because ^35^S-labeled full-length product is increased in ASCC-containing samples (e.g., Figure 4A), we suspected that the truncated peptidyl-tRNA species are disappearing because the ribosomes housing them elongate to the stop codon after ASCC removes the lead ribosome to relieve the roadblock.

**Figure 5:**
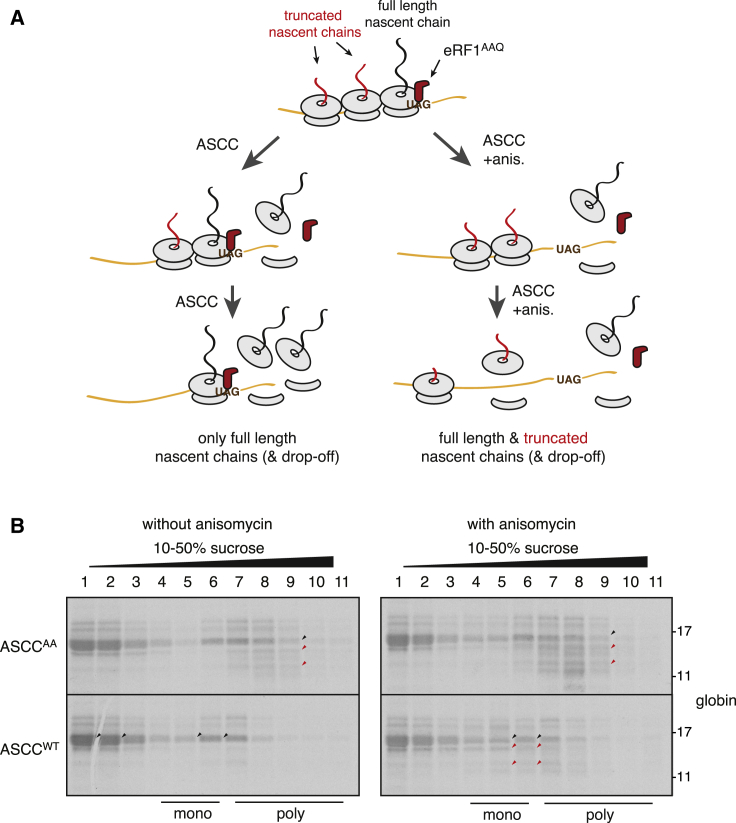
The Lead Ribosome of a Queue Is Targeted by ASCC (A) Expected results if ASCC targets only the lead ribosome in a queue in an ASCC-mediated disassembly reaction lacking or containing the elongation inhibitor anisomycin (anis.). (B) Results of the experiment depicted in (A) performed on eRF1^AAQ^-stalled collided polysomes. Black arrows point to full-length globins, and red arrows point to truncated nascent globins. As a control, the helicase-inactive mutant ASCC^AA^ was used instead of wild-type (WT) ASCC. Note the enrichment of truncated nascent globins in the monosome and subunit fractions with active ASCC only when elongation is inhibited (bottom right gel). Without anisomycin, active ASCC leads mostly to full-length products in the monosome and subunit fractions (bottom left gel). 50 nM pactamycin was used to inhibit initiation after 10 min, but similar results were seen without pactamycin. See also Figure S3.

To test this idea, we determined whether the disappearance of the truncated peptidyl-tRNA products could be blocked by a translation elongation inhibitor (Figure 5A). In this experiment, we produced the ribosome queue with eRF1^AAQ^ in the absence of ASCC, then added ASCC (or the helicase-dead ASCC^AA^) without or with the elongation inhibitor anisomycin. The truncated peptidyl-tRNAs produced by eRF1^AAQ^ remained in the polysome fraction with ASCC^AA^ but diminished with ASCC (Figure 5B, left). Anisomycin had no effect on the ASCC^AA^ reaction but stabilized the truncated products in the ASCC reaction (Figure 5B, right, red arrows). A sizable proportion of the truncated peptidyl-tRNAs stabilized by anisomycin shifted from the polysome fraction to the free and monosome fractions.

Similar conclusions could be drawn from experiments where we analyzed the disassembly of purified collided polysomes containing radiolabelled nascent chains in a system where additional radiolabel incorporation is not possible (Figure S3). Here, eRF1^AAQ^-stalled and ZNF598-ubiquitinated polysomes were isolated by centrifugation, then incubated with ASCC and post-ribosomal supernatant (S-100) from RRL. Focusing first on lipoxygenase, we found that S-100 alone did not disassemble polysomes (fractions 7–9) but ~50% disassembly (to fractions 4–6) was seen when ASCC was included. In case of globin, full-length nascent chains (black arrows) were partially redistributed from polysomes (fractions 7–9) to fractions 1–6, indicative of drop-off and subunit separation. Truncated nascent chains (red arrows) were not appreciably redistributed unless anisomycin was included with ASCC. This suggests that elongation factors in S-100 allow trailing ribosomes to escape disassembly by ASCC.

These findings make two important points. First, the lead ribosome is disassembled before the trailing ribosomes, allowing the latter to elongate once the roadblock is removed. Removing this roadblock strictly depends on the helicase activity of ASCC. Second, the trailing ribosomes only escape ASCC-mediated disassembly because they begin elongation. If elongation is inhibited, they are also potential targets for disassembly. The one exception might be the final ribosome, which would not be targeted by ASCC unless it had already been ubiquitinated by ZNF598 during the initial collision.

### ASCC Is Sufficient for Lead Ribosome Disassembly

**Figure 6:**
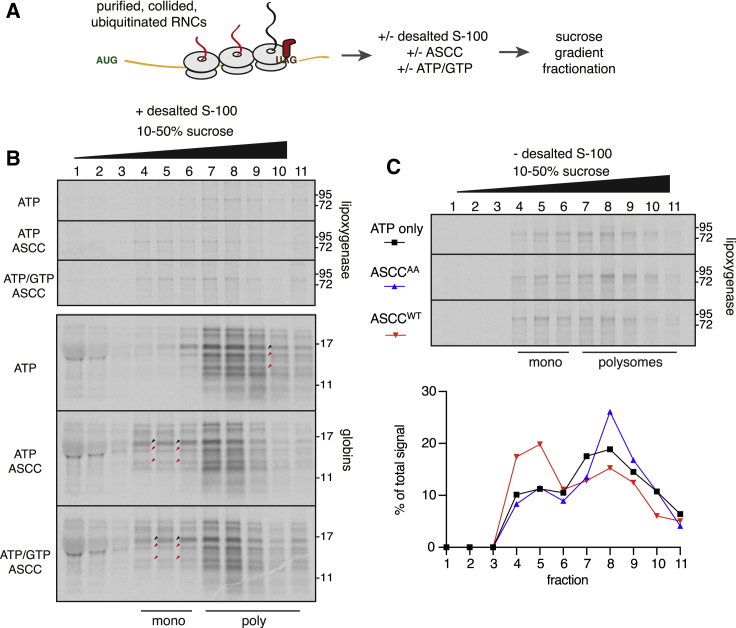
Reconstitution of ASCC-Mediated Ribosome Disassembly (A) Experimental strategy to test nucleotide requirements for ASCC-mediated ribosome disassembly. (B) Purified collided, ubiquitinated RNCs were incubated with ASCC in the presence of desalted S-100 and 0.5 mM of ATP and/or 0.5 mM GTP, and ribosome disassembly was analyzed by sucrose gradient fractionation. Nascent lipoxygenase (upper panel) and nascent globins (lower panel) were analyzed. Black arrows indicate full-length globins, and red arrows indicate truncated nascent globins. (C) The same RNCs as in (B) were incubated with WT ASCC or helicase-dead ASCC^AA^ and 0.5 mM ATP. Ribosome disassembly was monitored using the migration profile of the nascent lipoxygenase after sucrose gradient fractionation. The graph below shows relative density of the lipoxygenase band in each fraction as a percentage of the total signal. See also Figure S4.

Using the disassembly assay of purified collided polysomes, we tested the role of ATP versus GTP in the disassembly reaction (Figure 6A). The requirement for the helicase activity of ASCC3 and the apyrase experiment already established an ATP requirement, but the role of GTP was unclear. This was a key issue because the only known pathways for ribosome subunit separation involve GTPases. During termination, eRF1 is delivered by the GTPase eRF3, whereas splitting of stalled and empty ribosomes relies on Pelo delivery by the GTPase Hbs1L (Shao et al., 2016). Using desalted S-100, we found that addition of ASCC with ATP was sufficient to redistribute lipoxygenase (with ~50% efficiency) from the polysome to monosome and subunit fractions (Figure 6B, top panel). No additional stimulation was observed by including GTP in the reaction.

Similarly, no difference between reactions lacking or containing GTP was seen for globin nascent chains (Figure 6B, bottom panel). In this case, redistribution from the polysome to monosome and subunit fractions was seen not only for the full-length globin nascent chains (black arrows), but also for truncated products (red arrows). This can be explained because the desalted lysate is not elongation competent due to insufficient aminoacylated tRNAs. This result reinforces our conclusion that trailing ribosomes in the collided queue are targets for disassembly if they do not elongate when the leading ribosome roadblock is cleared.

Because anisomycin was not included in this experiment, we can exclude any potential artifacts stemming from its use. Furthermore, the trailing ribosomes are not engaged with eRF1^AAQ^, illustrating that ASCC-mediated disassembly does not rely on this particular method of stalling. Thus, ASCC can act on ribosomes stalled by anisomycin, eRF1^AAQ^, or aminoacyl-tRNA insufficiency, arguing that this mechanism is broadly applicable and does not rely on either subunit rotation state or occupancy status of the A-site.

The lack of GTP requirement suggests that GTPases, particularly the HbsL1-Pelo complex, are not needed for ASCC-mediated disassembly. To verify this conclusion in cells, we used acute knockdowns of either factor and analyzed the consequences for readthrough of a poly(A) stall in our dual-color reporter. Depletion of Hbs1L or Pelo did not impact terminal stalling at the poly(A) site (Figure S4A). Immunoblotting verified effective knockdowns and illustrated that Pelo was unstable in the absence of Hbs1L (Figure S4B).

Analysis of ribosome recycling of monosomes *in vitro* in the absence of ZNF598 and ASCC showed that recycling is only effective when there are few or no mRNA nucleotides in the A-site (Figures S4C and S4D). Thus, whereas a ribosome stalled at the end of a truncated mRNA is efficiently split by endogenous Pelo-Hbs1L, extension of the mRNA by 4 codons sharply prevents this reaction (Figure S4D). This is consistent with the observation that Pelo protrudes into the decoding center and would clash with mRNA there (Becker et al., 2011, Hilal et al., 2016, Shao et al., 2016). Although this reaction can clearly be forced using high levels of Pelo-Hbs1L *in vitro* (Shao et al., 2016), it does not appear to be effective at physiologic concentrations. Such methodological differences may explain somewhat discrepant observations in earlier reconstitution experiments (Pisareva et al., 2011, Shoemaker et al., 2010). An inability of the Pelo-Hbs1L system to efficiently recycle internal stalls would explain why a different recycling pathway (via ASCC) is needed in these circumstances.

During the course of our studies, we noted that the competency of stalled and collided polysomes to be recycled drops over time for reasons we don’t understand. For example, disassembling collisions as they occur by including ZNF598 and ASCC during the translation results in almost complete abrogation of queue formation (e.g., Figure 3D). By contrast, allowing queue formation followed by ASCC addition (e.g., Figure 3F) or using isolated polysomes followed by reconstitution with S-100 and ASCC (Figure S4) results in ~50% efficiency. In this context, we note that purified collided and ubiquitinated polysomes mixed with recombinant ASCC converted a subset (~30%) of polysomes into the monosome and subunit fraction as visualized by radiolabelled lipoxygenase nascent chains (Figure 6C). The specificity of this reaction was verified by the lack of effect with ASCC^AA^. These results indicate that ASCC uses its helicase activity to engage collided polysomes marked with ubiquitin by ZNF598 and disassembles the lead ribosome. Although we cannot exclude stimulatory factors, the ability of this reaction to proceed in a purified system indicates that ASCC is the minimal system for disassembly.

## Discussion

**Figure 7:**
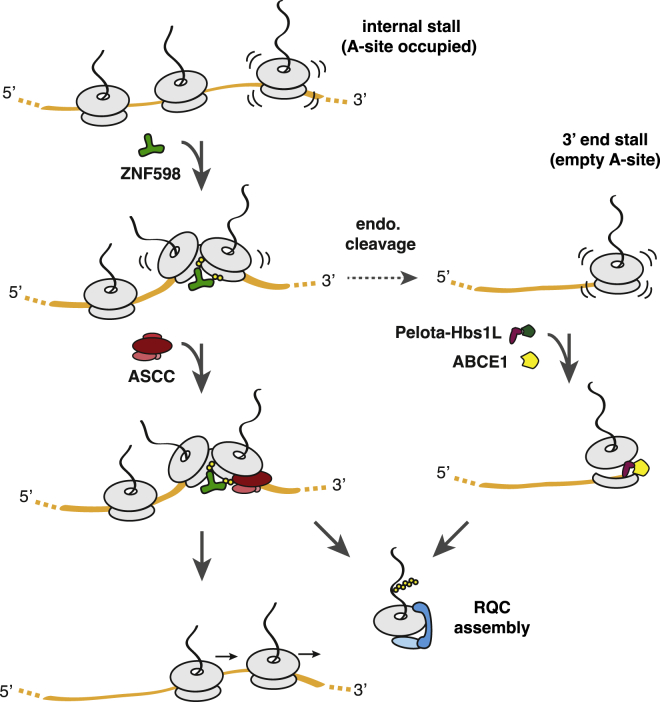
Pathways for the Resolution of Ribosome Stalls Ribosomes that stall internally within an mRNA (left) are recognized and resolved by a different mechanism than ribosomes that stall close to the 3′ end of an mRNA (right). With an internal stall, ribosome collision recruits the ubiquitin ligase ZNF598 to ubiquitinate 40S proteins. ASCC then acts on the lead ribosome to liberate a 60S-peptidyl-tRNA species that is targeted by RQC. The trailing ribosomes can then continue elongation. Without ASCC, collided ribosomes are subject to endonucleolytic cleavage between ribosomes to generate ribosome-nascent chain complexes that are now stalled at or close to the 3′ end of the mRNA (right). Such species are dissociated by Pelota-Hbs1L-ABCE1 to generate 60S-peptidyl-tRNA complexes that are engaged by RQC. See also Figure S5.

RQC engagement of a stalled ribosome strictly relies on removal of the 40S subunit (Shao et al., 2013) to expose binding sites for RQC factors (Lyumkis et al., 2014, Shao et al., 2015, Shen et al., 2015). Whereas a ribosome stalled at the 3′ end of an mRNA is an ideal substrate for the Pelo-Hbs1L complex (Shao et al., 2016), internally stalled ribosomes are not. Our study demonstrates that the recycling of internally stalled ribosomes is mediated by ASCC in an ATP-, ZNF598-, and ubiquitin-dependent reaction. The helicase activity of ASCC3, the core subunit of ASCC, is essential for recycling. Helicase-mediated ribosome splitting is entirely different from the previously known splitting reactions mediated by a translational GTPase complex (either eRF1-eRF3 or Pelo-Hbs1L) working with ABCE1. Thus, there are two qualitatively different mechanisms of disassembling a stalled ribosome: those at (or very close to) the 3′ end that rely on Pelo-Hbs1L and internal stalls that rely on ASCC (Figure 7).

The Pelo-Hbs1L system endogenous to RRL (Pisareva et al., 2011, Shao et al., 2013) does not rely on collisions, ZNF598, 40S ubiquitination, or ASCC. Conversely, the ASCC reaction does not require Pelo-Hbs1L but is strongly dependent on collision-specific 40S ubiquitination by ZNF598. Because a translating ribosome without mRNA in the A-site has no obvious physiologic correlate, this is necessarily an aberrant product that can be disassembled without risk of inappropriately aborting a functional translation complex. By contrast, a slow ribosome is a poor proxy for aberrant translation because there are physiologic contexts where this is beneficial (Pechmann et al., 2014, Stein et al., 2019, Yanagitani et al., 2011). Instead, the cell uses collisions as a more reliable proxy. This by itself is not sufficient and must be followed by a ubiquitination event, and the action of the ASCC before translation is aborted. Because we could reconstitute ASCC-mediated disassembly (albeit somewhat inefficiently) with purified collided ribosomes and recombinant ASCC, we believe this is the committed and irreversible step in aborting translation at an internal stall sequence.

The steps preceding ASCC are potentially reversible: collisions can be reversed if translation resumes and ubiquitinated 40S can be deubiquitinated (Garshott et al., 2020, Meyer et al., 2020). This means that translation is abandoned only when a collision is sufficiently long-lived to recruit ZNF598, and the 40S-ubiquitin mark lingers long enough to allow ASCC function. Multiple reversible checkpoints followed by a comparatively slower irreversible commitment step can facilitate enhanced discrimination of otherwise modest kinetic differences (Shao and Hegde, 2016, Shao et al., 2017, Zhang et al., 2013). Future kinetic studies of each step will be needed to fully understand the basis of target selection by the ZNF598-ASCC system.

Biochemical and structural analyses indicate that stalling at a poly(A) sequence is an intrinsic property of the ribosome-mRNA-polypeptide complex (Chandrasekaran et al., 2019). Structural studies of suboptimal di-codon stalls similarly suggest a ribosome-intrinsic mechanism (Tesina et al., 2020). Thus, it is likely that in cells lacking ZNF598 or ASCC, the extent of slowdown at such stalls (e.g., poly(A)) is unaffected. Yet, increased readthrough occurs despite stalling, albeit slowly and with poor fidelity (Garzia et al., 2017, Juszkiewicz and Hegde, 2017, Sundaramoorthy et al., 2017). Failure to disassemble ribosomes at this stall site in Slh1-lacking yeast explains the seemingly contradictory observations of increased footprints at the stall in ribosome profiling experiments (Sitron et al., 2017) despite near complete readthrough in functional assays (Matsuo et al., 2017).

Persistence of collisions at a stall site in Slh1-deficient yeast results in enhanced levels of mRNA cleavage via the putative nuclease Cue2 (D’Orazio et al., 2019). Ribosome profiling suggests that this cleavage occurs 5′ of the stalled ribosome, possibly near the A-site of the trailing ribosome (Guydosh and Green, 2017, Ikeuchi et al., 2019). Cleavage would therefore convert the trailing ribosome into an ideal substrate for rescue by Dom34-Hbs1. This might explain how these factors influence the fate of internally stalled ribosomes in some experimental paradigms (Doma and Parker, 2006, Passos et al., 2009, Tsuboi et al., 2012) but had no effect on our reporter in this study or minimal consequences genome-wide in ribosome profiling studies (Guydosh and Green, 2014). Very high expression of a stalling substrate might saturate Slh1-mediated rescue, favoring the Cue2-mediated pathway reliant on Dom34-Hbs1. Saturation might explain why a small molecule that stalls ribosomes on numerous mRNAs is dependent on both ASCC and Pelo-Hbs1L despite the stalls being internal (Liaud et al., 2019). The RRL system does not seem to recapitulate Cue2-like endonucleolytic cleavage of collided polysomes, explaining why disassembly of the lead ribosome allows trailing ribosomes to complete translation and why Pelo-Hbs1L would not participate in any aspect of collision resolution in RRL.

Although Pelo-Hbs1L is slow at rescuing internally stalled ribosomes, this reaction can occur over time or with high levels of the complex (Shao et al., 2013, Shao et al., 2015, Shoemaker et al., 2010). This might ensure that even without the ASCC pathway, ribosomes are not trapped indefinitely. Such a mechanism might explain how a stalled leading ribosome incapable of readthrough, which cannot be routed to the Dom34-Hbs1 pathway by Cue2, is eventually rescued in the absence of Slh1. Thus, what emerges is a picture where multiple rescue pathways with different specificities and mechanisms have sufficient overlap to provide robustness to the cell in avoiding ribosomes trapped on an mRNA (Figure 7).

The dependence on ZNF598 and ubiquitin point to ASCC acting specifically on collided ribosomes ubiquitinated on eS10 or uS10 (the targets of ZNF598 and Hel2, respectively). Consistent with this conclusion, mutation of these ubiquitination sites phenocopies ZNF598 or Hel2 deletion despite the presence of ASCC (Garzia et al., 2017, Juszkiewicz and Hegde, 2017, Matsuo et al., 2017, Sundaramoorthy et al., 2017). Ubiquitination specifically occurs on collided ribosomes (Ikeuchi et al., 2019, Juszkiewicz et al., 2018), although it is not yet clear whether ubiquitins are added to the leading, trailing, or both ribosomes. Regardless, our experiments suggest that the target for ASCC-mediated disassembly is the leading ribosome. Downstream ribosomes can elongate and escape ASCC after removal of the leading ribosome but are targeted for disassembly if elongation is impaired by either aminoacyl-tRNA insufficiency or an elongation inhibitor.

Although the requirement for 40S ubiquitination to abort translation at stalls is clear, the role played by ubiquitin remains mysterious. The simplest model, that the ubiquitin is used as a mark for ASCC recruitment, seems unlikely because ASCC interaction with ribosomes (Figure S5A) is unaffected in cells lacking ZNF598 (Figure S5B). Although other ubiquitins previously observed on ribosomes (Higgins et al., 2015, Ikeuchi et al., 2019) might recruit ASCC in the absence of ZNF598, this seems unlikely, given that ASCC cannot function without ZNF598 or the specific ubiquitins it adds to eS10 and uS10.

Mutation of the ubiquitin-binding CUE domain in ASCC2 has little or no functional consequence even though mutation of those same residues completely abolishes CUE-ubiquitin interactions and precludes ASCC recruitment to the ubiquitin-marked sites of DNA damage (Brickner et al., 2017). The earlier finding that a more severe mutation in this region subtly impacts ASCC function (Hashimoto et al., 2020) can be explained by our observation that this mutant protein is apparently less stable and expressed at lower levels in cells (Figure S5C). Even complete loss of ASCC2 only partially reduces the ASCC3-ribosome interaction (Figure S5B), consistent with a partial phenotype in readthrough assays (Figure 1D). Although we cannot entirely exclude a role for the CUE domain given that our rescue of ΔASCC2 cells were under overexpression conditions, its contribution would be modulatory and not essential. Thus, the mechanistic role of 40S ubiquitination remains to be elucidated.

Prior to its implication in ribosome-associated quality control, the main functions ascribed to ASCC were in responding to DNA repair in the nucleus (Brickner et al., 2017, Dango et al., 2011). This function appears to be independent of the cytosolic function because nuclear-specific factors that work with ASCC (such as ALKBH3) do not influence ribosome stalling in genetic studies (Liaud et al., 2019). Furthermore, the CUE domain is critical for the nuclear function but less central for disassembling collided ribosomes. Finally, ASCC1 and ASC-1 have no effect on ribosome disassembly under the conditions analyzed here even though they are a stable part of ASCC and associate with ribosomes. The physiologic relationship between the nuclear and cytosolic roles of ASCC, how these are regulated, and the functions of individual subunits in each role are interesting issues for the future.

The molecular basis of how ASCC uses its helicase activity to disassemble ribosomes remains to be dissected but is now feasible with a defined *in vitro* system. During the review of this paper, a study appeared showing that purified yeast RQT complex disassembles the lead ribosome of a stalled and collided tri-ribosome complex (Matsuo et al., 2020). As in our analysis, this reaction required 40S ubiquitination, ATP, and the ATPase activity of Slh1 but did not involve the Dom34/Hbs1/Rli1 splitting factors. Unlike the homologous helicase SKIV2L (Zinoviev et al., 2020), neither the ASCC nor the RQT complex extracts mRNA from arrested collided ribosomes. This is evident because trailing ribosomes can elongate after the leading ribosome is dispatched by ASCC and because disomes remain intact after the lead ribosome is disassembled by RQT (Matsuo et al., 2020). The reconstitution systems described here and by Matsuo et al. (2020) provide a foundation for future mechanistic studies in the mammalian and yeast systems, respectively.

## STAR★Methods

### Key Resources Table

**Table:** 

REAGENT or RESOURCE	SOURCE	IDENTIFIER
**Antibodies**		

Rabbit monoclonal anti-uL2	Abcam	Cat. #ab169538
Rabbit monoclonal anti-eS24	Abcam	Cat. #ab196652
Rabbit polyclonal anti-ASCC3	Bethyl	Cat. #A304-015A
Rabbit polyclonal anti-ASCC2	Bethyl	Cat. #A304-020A
Rabbit polyclonal anti-ASCC1	Bethyl	Cat. #A303-871A
Rabbit polyclonal anti-ASC-1	Bethyl	Cat. #A300-843A
Rabbit polyclonal anti-PELO	Bethyl	Cat. #A305-447A
Rabbit polyclonal anti-Hbs1L	Shao and Hegde, 2014	N/A
Mouse monoclonal anti-Flag	Sigma-Aldrich	Cat. #F3165 RRID:AB_259529
Rabbit polyclonal anti-GFP	Chakrabarti and Hegde, 2009	N/A
Rabbit polyclonal anti-RFP	Chakrabarti and Hegde, 2009	N/A
HRP conjugated mouse monoclonal anti-beta-Actin	Sigma-Aldrich	Cat. #A3854 RRID: AB_262011
HRP conjugated goat anti-rabbit	Jackson Immunoresearch	Cat. #111-035-003 RRID:AB_2313567
HRP conjugated goat anti-mouse	Jackson Immunoresearch	Cat. #115-035-003 RRID:AB_10015289

**Chemicals, Peptides, and Recombinant Proteins**		

3xFlag peptide	Sigma-Aldrich	Cat. #F4799
Anti-Flag M2 affinity resin	Sigma-Aldrich	Cat. #A2220
Ni-NTA agarose	QIAGEN	Cat. #30210
S7 Micrococcal Nuclease	Roche	Cat. #10107921001
Complete EDTA-free protease inhibitor cocktail	Roche	Cat. #11873580001
Pactamycin	Gift from E. Steinbrecher, Pharmacia	N/A
PYR-41 (E1 inhibitor)	Sigma-Aldrich	Cat. #N2915
Apyrase	New England Biolabs	Cat. #M0398
Hygromycin B	Millipore	Cat. #400051-100KU CAS: 31282-04-9
Blasticidin S	Santa Cruz Biotechnology	Cat. #sc204655 CAS: 3513-03-9
Anisomycin	Sigma-Aldrich	Cat. #A9789; CAS #22862-76-6
Doxycycline	Sigma-Aldrich	Cat. #D9891; CAS: 24390-14-5
EasyTag L-[^35^S]-Methionine	Perkin Elmer	Cat. #NEG709A005MC
CAP (diguanosine triphosphate cap)	New England Biolabs	Cat. #S1404L
RNasin	Promega	Cat. #N251
Amino acid kit	Sigma-Aldrich	Cat. #09416
SP6 Polymerase	New England Biolabs	Cat. #M0207L
Creatine phosphate	Roche	Cat. #621714
Creatine kinase	Roche	Cat. #127566
ZNF598-TEV-3xFlag (human)	Juszkiewicz and Hegde, 2017	N/A
eRF1-AAQ (human)	Brown et al., 2015	N/A
ASCC (human)	This paper	N/A

**Experimental Models: Cell Lines**		

HEK293T	ATCC	CRL-3216
ZNF598 KO Flp-In™ T-REx™ 293 dox inducible GFP-P2A-(K^AAA^)_21_-P2A-RFP	Juszkiewicz and Hegde, 2017	N/A
WT Flp-In™ T-REx™ 293 dox inducible GFP-P2A-(K^AAA^)_21_-P2A-RFP	Juszkiewicz and Hegde, 2017	N/A
WT Flp-In™ T-REx™ 293 dox inducible GFP-P2A-(K)_0_-P2A-RFP	Juszkiewicz and Hegde, 2017	N/A
ASCC3 KO Flp-In™ T-REx™ 293 dox inducible GFP-P2A-(K^AAA^)_21_-P2A-RFP	This paper	N/A
ASCC2 KO Flp-In™ T-REx™ 293 dox inducible GFP-P2A-(K^AAA^)_21_-P2A-RFP	This paper	N/A
Sf21 (*S. frugiperda*) insect cells	GIBCO	12682019
*E. coli* BL21(DE3) pLysS	Thermo Fisher	C606003

**Recombinant DNA**		

pcDNA3.1 ZNF598-TEV-3xFlag	Juszkiewicz and Hegde, 2017	N/A
pRSETA 6xHIS-TEV-eRF1(AAQ)	Brown et al., 2015	N/A
pcDNA3.1-ASCC3-3xFLAG	This paper	N/A
pcDNA3.1-ASCC3-3xFLAG-K505R	This paper	N/A
pcDNA3.1-ASCC3-3xFLAG-K1355R	This paper	N/A
pCMV6-MYKDDK-ASCC2	Origene	Cat. RC203391
pCMV6-MYKDDK-ASCC2-mut1, -mut2, and -mut3	This paper	N/A
pUCDM-ASCC2-FLAG-TRIP4	This paper	N/A
pFL-ASCC1-8His-ASCC3	This paper	N/A
pFL-ASCC1-8His-ASCC3(K505A/K1355A)	This paper	N/A
pX330-U6-Chimeric_BB-CBh-hSpCas9	Ran et al., 2013	Addgene Plasmid #42230

**Sequence-Based Reagents**		

gBlock – drop-off template: TCATACACATACGATTTAGGTGACACTATAGAA GCTTCTTGTTCTTTTTGCAGAAGCTCA GAATAAACGCTCAACTTTGGCAGATCTACC ATGGCGCCTGGTCCGACCCCCAGTGGCACT CAGATGGGATCCTCAGGGCGCTCTCCCAGCAA ATCTATGGCCGCCCGGGCGGCGGGATCCTCT ATGCGGCAGAGGAAAAATGCCTTATTAGGAT CCGACTACAAAGACCATGACGGTGATTATAAA GATCATGACATCGATTACAAGGATGACGATG ACAAGTAAGAATTCGTAATCATGTCATAGCTG	This study	N/A
Primer: gBlock 5′ Fwd: TCATACACATACGATTTAGG	Sharma et al., 2010	N/A
Primer: DO1 5′ Rev: TAAGGCATTTTTCCTCTGCCG	This study	N/A
Primer: DO2 5′ Rev: TAATAAGGCATTTTTCCTCTGCCG	This study	N/A
Primer: DO3 5′ Rev: TTTTTTTTTTAA TAAGGCATTTTTCCTCTGCCG	This study	N/A
Primer: DO4 5′ Rev: TTTTTTTTTTTTTAAT AAGGCATTTTTCCTCTGCCG	This study	N/A
Primer: DO5 5′ Rev: GTCGGATCCTAA TAAGGCATTTTTC	This study	N/A
Primer: DO6 5′ Rev: GTAGTCGGAT CCTAATAAGGC	This study	N/A
Primer: K1355A site directed mutagenesis primer 5′Fwd GTCCTACTTGGAGCACCTACTGGATCG GGAGCCACTGTTGCAGCTGAATTAGCC	This study	N/A
Primer: K505A site directed mutagenesis primer 5′Fwd GTGCCCCTACAGGAGCTGGAGCAACCA ACATTGCAATGCTGACAGTCTTGC	This study	N/A
Primer: ASCC2 in pUCDM Fwd CATCGGGCGCGGATCCATGCCAGCTCT GCCCCTGGAC	This study	N/A
Primer: ASCC2 in pUCDM Rev TTCGGACCGGGATCCTCACTTGTCGTCATCGTC TTTGTAGTCGGATGGGATCATGCCTTTGCTC	This study	N/A
Primer: TRIP4 in pUCDM Fwd CACCCGGGATCTCGAGATGGCGGTGGCTGG GGCGGTG	This study	N/A
Primer: TRIP4 in pUCDM Rev AGCACCATGGCTCGAGTTAGACAGCTTTAT TCTGCTTCAT	This study	N/A
Primer: ASCC3 in pFL Fwd CACCCGGGATCTCGAGATGGCTTTACCTCG TCTCACAG	This study	N/A
Primer: ASCC3 in pFL Rev AGCACCATGGCTCGAGTTACTTTAATGCCAG GTCAGTCAGG	This study	N/A
Primer: ASCC1 in pFL Fwd CATCGGGCGCGGATCCATGGAAGTTCTG CGTCCACAG	This study	N/A
Primer: ASCC1 in pFL Rev CTTCGGACCGGGATCC TCAGTGGTGGTGGTGGTGGTGGTGGTGGGA GAAGTCAATTTGTCCACA	This study	N/A
*i*e-1 specific primer 5′ Fwd: CCCGTAACGGACCTCGTACTT	This study	N/A
*i*e-1 specific primer 5′ Rev: TTATCGAGATTTATTTGCATACAACAAG	This study	N/A
Silencer Select Pre-designed siRNA against ASCC3 #1	Life Technologies	siRNA ID #s21603
Silencer Select Pre-designed siRNA against ASCC3 #2	Life Technologies	siRNA ID #s21604
Silencer Select Pre-designed siRNA against ASCC2 #1	Life Technologies	siRNA ID #s38590
Silencer Select Pre-designed siRNA against ASCC2 #2	Life Technologies	siRNA ID #s23588
Silencer Select Pre-designed siRNA against ASCC1 #1	Life Technologies	siRNA ID #s27225
Silencer Select Pre-designed siRNA against ASCC1 #2	Life Technologies	siRNA ID #s27224
Silencer Select Pre-designed siRNA against ASC-1 #1	Life Technologies	siRNA ID #s17820
Silencer Select Pre-designed siRNA against ASC-1 #2	Life Technologies	siRNA ID #s17822
guide RNA targeting exon 1 of ASCC3 gene 5′- TTTTAGATTTGGGCCTGACA-3′	This study	N/A
guide RNA targeting exon 2 of ASCC3 gene 5′-GAAGGACTCTGTTGGTCACA-3′	This study	N/A
guide RNA targeting exon 2 of ASCC2 gene 5′ – TGTCCCCCGCAAATTCGACG- 3′	This study	N/A

**Software and Algorithms**		

FlowJo	FlowJo, LLC	https://www.flowjo.com/
GraphPad Prism	GraphPad Software	https://www.graphpad.com/
Adobe Illustrator	Adobe	https://www.adobe.com/

**Other**		

SuperSignal West Pico Chemiluminescent substrate	Thermo Fisher	Cat. #34080
Rabbit Reticulocyte Lysate Mix	Sharma et al., 2010	N/A
DMEM, high glucose, GlutaMAX™ Supplement, pyruvate	Thermo Fisher	Cat. #10569010
Tetracycline-free Fetal Calf Serum (FCS)	BioSera	Cat. #FB-1001T/500
Lipofectamine RNAiMAX	Thermo Fisher	Cat. #13778150
TransIt 293	Mirus	Cat. #MIR 2705
PonceauS Solution	Sigma-Aldrich	Cat. P-7170-1LCAS: 6226-79-5
QuikChange Lightning Multi Site-Directed Mutagenesis Kit	Agilent Technologies	Cat. #2100515-5
MAX Efficiency DH10Bac	GIBCO	Cat. 10361-012
2-L Tissue Culture Roller Bottles	Biofil	Cat. TCB-012-002
Sf-900 SFM (1x) Serum Free Medium Complete	GIBCO	Cat. 12658-027
Basemuncher	Expedeon	Cat. #BM0100
Heparin Sepharose CL-6B	GE Healthcare	Cat. 17-0552-02
In-Fusion HD Cloning Kit	Takara Bio	Cat. 121416
BamHI-HF	NEB	Cat. R3136S
XhoI	NEB	Cat. R0146S
Cre recombinase	NEB	Cat. M0298S

### Resource Availability

#### Lead Contact

Further information and requests for resources and reagents should be directed to and will be fulfilled by the Lead Contact Ramanujan S. Hegde (rhegde@mrc-lmb.cam.ac.uk).

#### Materials Availability

Plasmids, custom antibodies, and cell lines are available from the authors upon request.

#### Data and Code Availability

No datasets or code are associated with this paper.

### Experimental Model and Subject Details

#### Cell lines

HEK293-based mammalian cells used in this study were cultured in Dulbecco’s Modified Eagle’s Medium (DMEM) with 10% tetracycline-free fetal calf serum (FCS). Stable cell lines containing doxycycline-inducible reporters were supplemented with 15 μg/mL blasticidin and 100 μg/mL hygromycin. Parental HEK293 Flp-In Trex cells with stably integrated (K^AAA^)_21_ and (K)_0_ fluorescent reporters were previously described (Juszkiewicz and Hegde, 2017). For CRISPR-Cas9 mediated knockout of ASCC2 and ASCC3 in the (K^AAA^)_21_ reporter cell line, guide RNAs targeting exons 1 and 2 of ASCC3 (5′- TTTTAGATTTGGGCCTGACA-3′; 5′-GAAGGACTCTGTTGGTCACA-3′ respectively), and exon 2 of ASCC2 (5′ – TGTCCCCCGCAAATTCGACG- 3′) were designed using the CRISPR design tool at http://zlab.bio/guide-design-resources cloned into the px330-U6 plasmid (Ran et al., 2013), and used to generate knockout cells as described (Juszkiewicz and Hegde, 2017). Single-cell derived clones were screened for the gene disruption by western blotting. Usually multiple knockout clones per each guide RNA were isolated and the phenotypes were verified to be the same among them using flow cytometry fluorescent reporter assays. For the induction of stably integrated transgenes, cells were treated with 1 μg/mL of doxycycline for 20 h. All transient transfections were performed with TransIt 293 reagent from Mirus. siRNA silencing was for 3 days using RNAiMAX (Thermo Fisher) according to manufacturer’s protocol. Silencer select siRNAs were from Thermo Fisher and details are available in the key resources table. Cell lines were routinely checked for mycoplasma contamination and verified to be negative. Identity of the cell lines was verified by antibiotic resistance markers distinctive to HEK293 Flp-In Trex cells and reporters integrated into the unique FRT site.

### Method Details

#### Constructs and antibodies

ASCC3 for mammalian expression was in the pcDNA3.1 vector containing a C-terminal 3xFLAG. The ASCC3 ORF was PCR amplified from a commercially available plasmid (Origene, Cat. No RC216672) and sub-cloned into the pcDNA-3xFLAG backbone. K505R and K1355R mutants of ASCC3 were generated using site-directed mutagenesis. FLAG-tagged ASCC2 for mammalian expression was obtained from Origene (Cat. No RC203391). ASCC2-mut1 contained three point mutations within the LLP motif of the CUE domain: L478A, L479A, P480A. These mutations abolish interaction with ubiquitin. ASCC2-mut2 contained the same mutations as ASCC2-mut1 plus V487A and L491A. ASCC2-mut3 was designed to bind ubiquitin stronger than WT and contained two point mutations L478M and L479F. ASCC genes were assembled into a multigene cassette for ASCC expression in insect cells (Fitzgerald et al., 2006) as follows. ASCC2 with a C-terminal FLAG tag and untagged ASC-1 were cloned into BamHI and XhoI sites, respectively, of the pUCDM donor vector using the In-Fusion HD Cloning Kit (Takara Bio). ASCC1 with a C-terminal 8-histidine tag and untagged ASCC3 were cloned into BamHI and XhoI sites, respectively, of the pFL acceptor vector using the In-Fusion HD Cloning Kit. After verifying all inserts by sequencing, pUCDM containing ASCC2 and ASC-1 was fused with pFL containing ASCC1 and ASCC3 by *in vitro* Cre-Fusion. ASCC^AA^ containing the K505A and K1355A mutations was generated using the QuikChange Lightning Multi Site-Directed Mutagenesis Kit (Agilent Technologies) on pFL containing ASCC3 and ASCC1. Mutagenesis was verified by sequencing. Antibodies against ASCC were from Bethyl Laboratories. The details about these and other antibodies are provided in the key resources table.

#### Flow cytometry

Flow cytometry analysis of the fluorescent reporters was performed as described (Juszkiewicz and Hegde, 2017). The dual color reporter (see Figure 1A) is stably integrated into the single FRT site of HEK293 Flp-In Trex cells. The reporter is driven by a doxycycline-inducible promoter. Approximately ~20 h after induction of the fluorescent reporters, cells were washed with PBS, trypsinized, resuspended in DMEM containing 10% FCS, spun for 3 min at 5000 rpm in a tabletop centrifuge and resuspended in ice cold PBS. Data was collected using LSRII instrument (Becton Dickinson) and analyzed in FlowJo software. In each experiment, at least ~20,000 GFP positive events were analyzed. No other gating was employed. The RFP:GFP ratio was plotted as a histogram. A lower RFP:GFP ratio indicates increased stalling. The histograms within any graph are directly comparable because the data were collected at the same time with the same detector settings. The ratios between graphs are approximately, but not precisely comparable due to some variations in detector efficiencies and settings on different days.

#### Western Blot analysis

For analysis of total cellular proteins, cells were washed with PBS prior to lysis with 100 mM Tris pH 8.0 with 1% SDS. Cell lysates were heated for 10 min at 95°C with vortexing to shear genomic DNA. After adjusting protein concentrations of the samples based on A_280_ values, 5xSDS sample buffer (250 mM Tris, 5%SDS, 50% glycerol and 500 mM DTT) was added to a final concentration of at least 1x. For the analysis of samples after sucrose gradient centrifugation, each fraction from the gradient was adjusted with 5xSDS sample buffer and analyzed directly by electrophoresis. Electrophoresis employed 9% and 12% Tris-Tricine-based gels. After electrophoresis, proteins were transferred to 0.2 μm nitrocellulose membrane. Blocking and antibody incubations were typically for 1 h at room temperature with 5% nonfat powdered milk in PBS containing 0.1% Tween-20 (PBS-T). In some experiments, primary antibodies were incubated overnight at 4°C. Detection employed HRP-conjugated secondary antibodies and SuperSignal West Pico Chemiluminescent Substrate (Thermo Fisher).

#### *In vitro* translation

*In vitro* translation of endogenous mRNAs in rabbit reticulocyte lysate was performed as described (Juszkiewicz et al., 2018). Typically, translation reactions contained 33% of crude rabbit reticulocyte lysate (Green Hectares), 20 mM HEPES, 50 mM K(OAc), 2 mM MgCl_2_, 10 mM KOH, 40 μg/mL creatine kinase, 12 mM creatine phosphate, 20 μg/mL pig liver tRNA, 1 mM ATP, 1 mM GTP, 1 mM reduced glutathione, 0.3 mM spermidine and 40 uM of each amino acid. Where indicated, methionine was omitted and replaced with 0.5 μCi/μl ^35^-S-methionine in order to radiolabel nascent polypeptides. Where indicated in the figure legends, purified eRF1^(AAQ)^ was used at 0.8 μM, purified 3xFLAG-ZNF598 was used at 75 nM and purified ASCC was added to 50 nM final concentration. Earlier studies have shown that 0.5 μM eRF1^(AAQ)^ competes with endogenous eRF1 at ~30%–40% (Brown et al., 2015). Thus, in our experiments, competition is somewhat better (perhaps ~60%–80%) but not complete, which is why some termination is observed even in reactions containing eRF1^(AAQ)^. The proteins were added either at the beginning of the reaction or after 45 min of elongation, as indicated on the individual figures. Translations were incubated for 45 min at 32°C in a water bath. When indicated, pactamycin was added after 10 min incubation at 32°C to 0.05 μM final concentration to ensure complete inhibition of translation initiation. Apyrase was used at 12.5 U/mL for 45 min at 32°C. Reactions containing 330 μM PYR-41 were initiated without ZNF598 and ASCC, which were added after 15 min to allow inhibition of E1 and discharge of pre-charged E2’s.

#### Purification of ribosome-nascent-chains

50 μl of translation reaction containing 0.8 μM eRF1^(AAQ)^, 75 nM ZNF598, and ^35^-S-methionine was incubated for 45 min at 32°C, chilled on ice, adjusted to 750 mM K(OAc) and 10 mM Mg(OAc)_2_ and layered atop of 200 μl of 20% sucrose cushion in high salt buffer (50 mM HEPES pH 7.6, 750 mM K(OAc), 10 mM Mg(OAc)_2_) in an ultracentrifuge tube. After centrifugation at 100,000 rpm at 4°C in the TLA120.2 rotor, the supernatant was aspirated, ribosomal pellets were washed with 1xRNC buffer [50 mM HEPES, pH 7.6, 100 mM K(OAc), 5 mM Mg(OAc)_2_] and resuspended in 25 μl of 1xRNC buffer on ice.

#### Ribosome disassembly reactions

Purified radiolabelled ribosome-nascent-chain complexes (RNCs) prepared as described above were used at ~50 nM (absorbance of 2.5 at 260 nm) in disassembly reactions. Where indicated in the figure legends, the reactions contained 50 nM ASCC (or a matched buffer), 45% (by volume) post-ribosomal S-100 of crude reticulocyte lysate (see below), 0.5 mM ATP, and 0.5 mM GTP, and 0.8 μM eRF1^(AAQ)^. In reactions without S-100, the final concentration of K(OAc) was adjusted to 250 mM to minimize precipitation of ASCC. When indicated, anisomycin was used at 50 μM concentration to inhibit translation elongation. Disassembly reactions were incubated for 45 min at 32°C water bath. S-100 was prepared by centrifugation of crude reticulocyte lysate at 100,000 rpm at 4°C for 1 h. Where indicated, desalted S-100 was prepared from S-100 by passage over a PD-10 desalting column, being careful to collect only the peak fractions (easily identifiable by the bright red color of haemoglobin) to minimize any dilution.

#### Sucrose gradient fractionation

20 μl of *in vitro* translation reactions or ribosome disassembly reactions were prechilled on ice and loaded atop of 200 μl of 10%–50% sucrose gradients in 220 μl centrifugation tubes. The gradients were prepared by successively layering 40 μl each of 50%, 40%, 30%, 20% and 10% sucrose in 1xRNC buffer and allowing to equilibrate for ~1 h. Unless otherwise noted, centrifugation was in the TLS-55 rotor (with suitable tube adaptors) at 55 000 rpm with the slowest acceleration and deceleration settings for 20 min at 4°C. Eleven fractions, 20 μl each, were collected manually from the top of the gradient. Based on immunoblotting for uL2 and eS24 (e.g., Figure 3C), these spin conditions result in subunits and 80S ribosomes in fractions 4-6 (labeled ‘mono’ in the figures) and polysomes in fractions 7-10 (labeled ‘poly’ in the figures). This separation is exceptionally reliable as routinely validated by uL2 and eS24 blots. To save space, only the eS24 blot is shown in most instances. Where indicated, the samples were treated with 0.1 mg/mL RNase A for 30 min at 37°C. The samples were mixed with 5xSDS sample buffer and analyzed directly by electrophoresis. For the analysis of the tRNA-attached nascent chain species, samples were not treated with RNase A and were analyzed by 12% Bis-Tris based gels run in MES-SDS running buffer. Samples treated with RNase A were analyzed by Tris-Tricine based system using 15% gels to resolve truncated nascent globins.

#### ASCC interaction with ribosomes in cells

Usually, two 10 cm plates of cells at around 80% confluency were used for each genotype (either WT, ASCC3 KO, ASCC2 KO or ZNF598 KO). Cells were first washed with ice-cold PBS and harvested by scraping. After sedimentation at 4°C at 5000 rpm for 3 min, cell pellets were resuspended in 200 μL of 1xRNC buffer containing 40 U/mL of RNAsin (Promega), 0.01% digitonin, 1x protease inhibitor cocktail (EDTA-free cOmplete from Roche) and 1 mM DTT. After 15 min incubation on ice, cells were disrupted using a pre-chilled 26G needle appended to 1 mL syringe. Lysates were clarified by 15 min centrifugation at 15,000 g at 4°C in a tabletop centrifuge. Concentrations of the lysates were adjusted to between 75-150 μg/mL (depending on the experiment) in 20 μL volume, loaded on a 10%–50% analytical sucrose gradients (200 μl) prepared as described above and spun for 30 min at 55,000 rpm in TLS-55 rotor at 4°C using slowest acceleration and deceleration settings. Eleven fractions of 20 μl were collected manually from the top of the gradient.

#### Drop-off assay for ribosome splitting

A synthetic gBlock ordered from IDT served as a universal template for PCR amplification of different drop-off constructs (DO1-DO6). The forward primer annealed at the 5′ end of the gBlock and reverse primers annealing near the 3′ end of the gBlock contained additional test sequences as described in figure legend. Sequences of the gBlock and primers are available in key resources table. PCR products were purified using PCR purification kit (QIAGEN). *In vitro* transcription was as described previously (Sharma et al., 2010). Briefly, transcription reaction used purified PCR product at 5 ng/μl in 40 mM HEPES pH 7.4, 6 mM MgCl_2_, 20 mM spermidine, 10 mM reduced glutathione, 0.5 mM ATP, 0.1 mM GTP, 0.5 mM UTP, 0.5 mM CTP, 0.5 mM Cap analog, 0.4-0.8 U/μl RNasin and 0.4 U/μl SP6 polymerase. Transcription reactions were incubated for 1 h at 37°C water bath. Transcription reaction was used directly in the *in vitro* translation reactions constituting 5% of the reaction by volume. *In vitro* translation reaction was as described above, except that crude RRL was pre-treated with micrococcal nuclease to digest endogenous mRNAs as described before (Sharma et al., 2010), and was not supplemented with exogenous tRNA. This was to ensure ribosomal stalling on UUAUUA leucine di-codons, which are poorly decoded due to a shortage of the appropriate tRNA in rabbit reticulocyte lysate (Feng and Shao, 2018). Translation reactions were incubated for 30 min at 32°C water bath. After chilling on ice, each reaction (20 μl) was loaded on a 10%–50% analytical sucrose gradient (200 μl) prepared as described above and spun for 20 min at 55 000 rpm in TLS-55 rotor at 4°C with slowest acceleration and deceleration settings. Eleven fractions, 20 μL each, were collected manually from the top of the gradient, fractions 1-4, 5-8 and 9-11 were pooled together, adjusted with 5xSDS sample buffer and analyzed using Bis-Tris gels run in MES-SDS buffer to ensure preservation of tRNA-nascent polypeptide species.

#### Purification of eRF1^(AAQ)^

The plasmid pRSETA 6xHIS-TEV-eRF1^(AAQ)^ (Brown et al., 2015) was transformed into BL21 (DE3) *E. coli* strain and plated onto a 10 cm agar plate. The next day, 120 mL of LB was inoculated with 1/3 of the bacterial lawn from the plate, grown for 3.5 h at 37°C with shaking, and added to 2L of fresh LB. After another ~2 h of growth at 37°C with shaking, when the culture reached ~0.6 OD_600_, 0.2 mM IPTG was added and growth was continued for 2 h at 37°C. Bacterial cultures were spun for 30 min at 6000 rpm in a JA8.1 rotor at 4°C. Cell pellets were washed with ice-cold PBS, spun again for 25 min at 3800 rpm and pellets were flash frozen in liquid nitrogen. After thawing in a room temperature water bath, the bacteria were resuspended 40 mL lysis buffer [1xPBS, 250 mM NaCl, 10 mM imidazole, 1 mM DTT, 1x complete protease inhibitor cocktail (Roche)] and sonicated. Lysates were spun for 40 min at 18000 rpm in JA25.50 rotor at 4°C and the supernatant was passed through a 1 mL column of pre-equilibrated NiNTA resin. The column was washed with 50 mL of lysis buffer and eluted three times with 1 mL of elution buffer (1xPBS, 250 mM NaCl, 250 mM imidazole, 1 mM DTT). The first two elution fractions (2 mL) were pooled together and dialysed overnight against 1L of 50 mM HEPES, 250 mM KOAc, 5 mM Mg(OAc)_2_, 10 mM imidazole, 10% glycerol, 1 mM DTT and TEV protease (added to achieve a 1:50 ratio of protease:protein). The dialysate was passed again over a fresh 1 mL column of NiNTA resin and aliquots of the flow-through were flash frozen in liquid nitrogen.

#### Purification of ZNF598

ZNF598-3xFLAG was purified from HEK293T as described previously (Juszkiewicz and Hegde, 2017). Briefly, each of the four 10 cm plates of HEK293T cells was transfected with 10 μg of pcDNA3.1-ZNF598-TEV-3xFLAG plasmid. After 24 h, each plate was split 1:4 and expression of the recombinant protein was allowed for another 48 h. Then, sixteen confluent plates were washed with ice-cold PBS, collected in ice-cold PBS by scraping and spun for 5 min at 3000 rpm. The wash with PBS was repeated once again and cells were lysed for 15 min on ice in 1 mL of lysis buffer [50 mM HEPES pH 7.6, 100 mM K(OAc), 5 mM Mg(OAc)_2_, 1 mM DTT, 1x complete protease inhibitor cocktail (Roche), 0.01% digitonin]. Complete cell lysis was ensured by 15 passes of the lysate through 26G needle attached to 2 mL syringe. The lysate was spun for 10 min at maximum speed in tabletop centrifuge at 4°C and supernatant was incubated with 100 μl of anti-FLAG M2 resin (Sigma) for 1 h in cold room with end-over-end rolling. The beads were first washed three times with lysis buffer, then 3 times with lysis buffer containing 400 mM K(OAc) and finally three times with 1xRNC buffer. Elution was for 20 min at room temperature with 100 μl of 1xRNC buffer containing 0.2 mg/mL FLAG peptide. Two consecutive elutions were performed and combined. The final concentration of the purified protein in 200 μl elution volume was typically ~2-4 μM.

#### Purification of ASCC

Baculovirus containing either wild type or double mutant ASCC was generated and amplified as described previously (Fitzgerald et al., 2006). Briefly, the isolated bacmids were transfected into Sf21 (*S. frugiperda*) insect cells using Mirus *Trans*IT®-Insect Reagent according to manufacturer’s instructions to obtain the initial virus (V_0_). V_0_ was then serially passaged to obtain V_1_ and V_2_ in Sf21 cells grown at 27°C, shaking at 80 rpm. The viral titer was determined using a qPCR-based method with primers designed to target the baculovirus essential gene *i*e-1 (Lo and Chao, 2004). 4 L of Sf21 insect cells at 0.5 × 10^6^ cells/mL were cultured at 27°C, shaking at 130 rpm, in Sf-900 III serum free medium (GIBCO) using 2-L shaker flasks containing 400 mL insect culture in each flask. The baculovirus containing either wild type or double mutant ASCC construct(s) was added at a multiplicity of infection of 1. The insect cells were harvested 3 days post-infection using a JS 4.2 rotor (Beckman Coulter J6-MI centrifuge) at 2500 rpm, 4°C for 15 min and re-suspended in 150 mL ASCC lysis buffer [20 mM Tris-HCl 8.0, 150 mM NaCl, 10% glycerol, 20 mM imidazole, 2.5 mM MgCl_2_, complete protease inhibitor cocktail (Roche), and 2500 units of Basemuncher (Expedeon) per liter of insect cell culture]. The pellet was homogenized with a dounce tissue grinder and sonicated at 50% amplitude (Branson digital sonifier) for 5 min in ice-cold water. The lysate was centrifuged at 40000 rpm in a Type 45 Ti rotor (Beckman Coulter Optima™ L-100 XP ultracentrifuge) for 1 h at 4°C. The supernatant was collected and incubated with 5 mL NiNTA resin (QIAGEN) (pre-equilibrated with 5 column volumes of ASCC lysis buffer) for 3 h at 4°C with end-over-end rolling. The resin was washed with 20 column volumes ASCC wash buffer [20 mM Tris-HCl 8.0, 300 mM NaCl, 10% glycerol, 20 mM imidazole, complete protease inhibitor cocktail (Roche)] and five sequential elutions were performed, each using 5 mL NiNTA elution buffer [20 mM Tris-HCl 8.0, 300 mM NaCl, 10% glycerol, 200 mM imidazole and complete protease inhibitor cocktail (Roche)]. The pooled eluate was incubated with anti-FLAG M2 resin (Sigma) pre-equilibrated with 5 mL ASCC wash buffer with end-over-end rolling at 4°C overnight. The anti-FLAG M2 resin was washed with 3 × 20 mL ASCC wash buffer without imidazole followed by an additional wash with M2 wash buffer [20 mM Tris-HCl 8.0, 150 mM NaCl, 10% Glycerol and complete protease inhibitor cocktail (Roche)] at 4°C. Elution was performed 5 times with 700 μL M2 elution buffer [20 mM Tris-HCl 8.0, 150 mM NaCl, 10% Glycerol, 1 μg/μl of FLAG peptide and complete protease inhibitor cocktail (Roche)]. The pooled eluate was passed through 200 μL of heparin Sepharose resin (GE healthcare) that was pre-equilibrated with 5 mL heparin wash buffer [20 mM Tris-HCl 8.0, 150 mM NaCl, 10% glycerol, 1 mM DTT and complete protease inhibitor cocktail (Roche)] at 4°C and washed with 5-column volumes heparin wash buffer at 4°C. Elution was performed five times with 200 μL each of heparin elution buffer [20 mM Tris-HCl 8.0, 400 mM NaCl, 10% glycerol, complete protease inhibitor cocktail (Roche) and 1 mM DTT] at 4°C. The final concentration of purified protein in a 200 μL elution volume was typically around 0.2-0.5 mg/mL.

### Quantification and Statistical Analysis

For quantification of the autoradiography signal, radioactive gels were exposed to phosphor imager and scanned using Typhoon instrument (GE Healthcare). Densitometry was performed using Fiji software and graphs were plotted using Graphpad (Prism). No statistical analyses were performed in this study. Reproducibility was ensured because each result shown in the paper is representative of at least two fully independent experiments with the same outcome. Two independent recombinant purified ASCC preparations were used in the course of this study and showed indistinguishable results. In addition to replication, each siRNA knockdown result was verified for two independent siRNA sequences. Results with the translation inhibitor anisomycin were verified with the unrelated translation inhibitor didemnin B (not shown in the paper).

## Supplemental Information

Document S1. Figures S1–S5

Document S2. Article plus Supplemental Information
