# Supplementary material for: The ASC-1 Complex Disassembles Collided Ribosomes
Source: Mol Cell. 2020 Aug 20;79(4):603–614.e8. doi: 10.1016/j.molcel.2020.06.006 (PMC7447978; doi:10.1016/j.molcel.2020.06.006)
Supplement: Document S1. Figures S1–S5 [file mmc1.pdf]

Molecular Cell, Volume *79*

Supplemental Information

The ASC-1 Complex Disassembles Collided Ribosomes

Szymon Juszkiewicz, Shaun H. Speldewinde, Li Wan, Jesper Q. Svejstrup, and Ramanujan S. Hegde

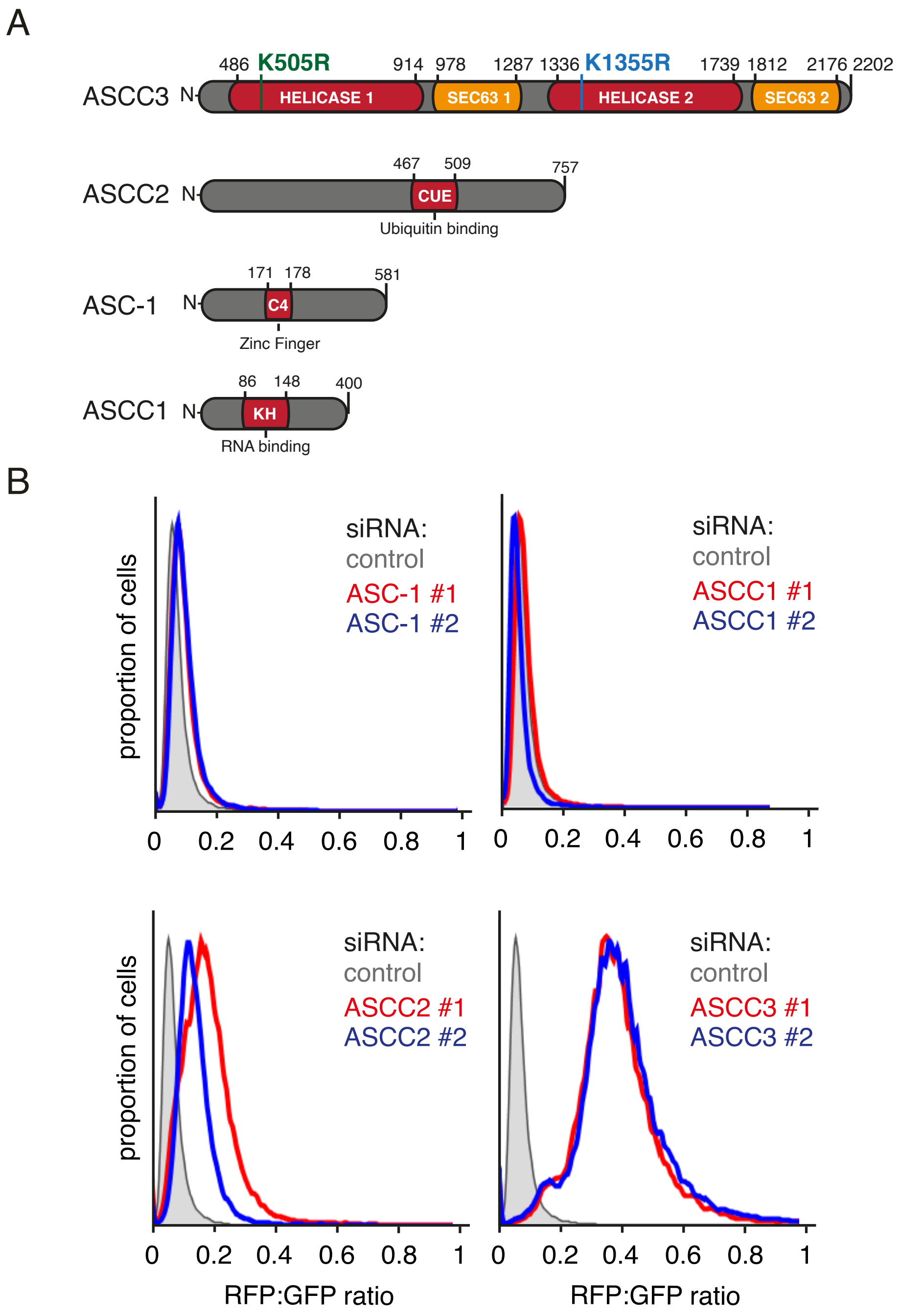

Figure S1. Characterization of the ASC-1 complex. Related to Figure 1.
(A) Schematic representation of the domain architecture of individual components of the ASC-1 complex (ASCC). K505R mutation is marked in green and K1355R mutation is marked in blue. These mutations inactivate the N- and C-terminal helicase domains, respectively. In experiments with recombinant ASCC, these same residues were mutated to alanines to produce a helicase-inactive complex. **(B)** WT $(K^{AAA})_{21}$ cells were treated with control (gray shaded) or two indepenedent siRNA sequences targeting ASCC components for 72h before induction of the stably integrated reporter with doxycycline for 20h. The RFP:GFP ratio as determined by flow cytometry was plotted as a histogram. A subset of these data (displaying only one of two siRNAs for each target) is presented in Figure 1D.

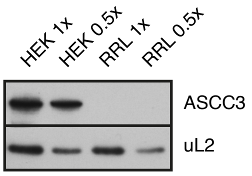

Figure S2. Immunoblotting of ASCC3 in RRL versus HEK293 cells. Related to figure 3.
Dilutions of total cell lysates from HEK293 cells and rabbit reticulocyte lysate (RRL) were normalised to equal amounts of ribosomes and analysed by western blotting using antibody against endogenous ASCC3. The absence of ASCC3 by blotting matches the finding that no collided ribosome disassembly is observed in RRL unless it is supplemented with exogenous ASCC.

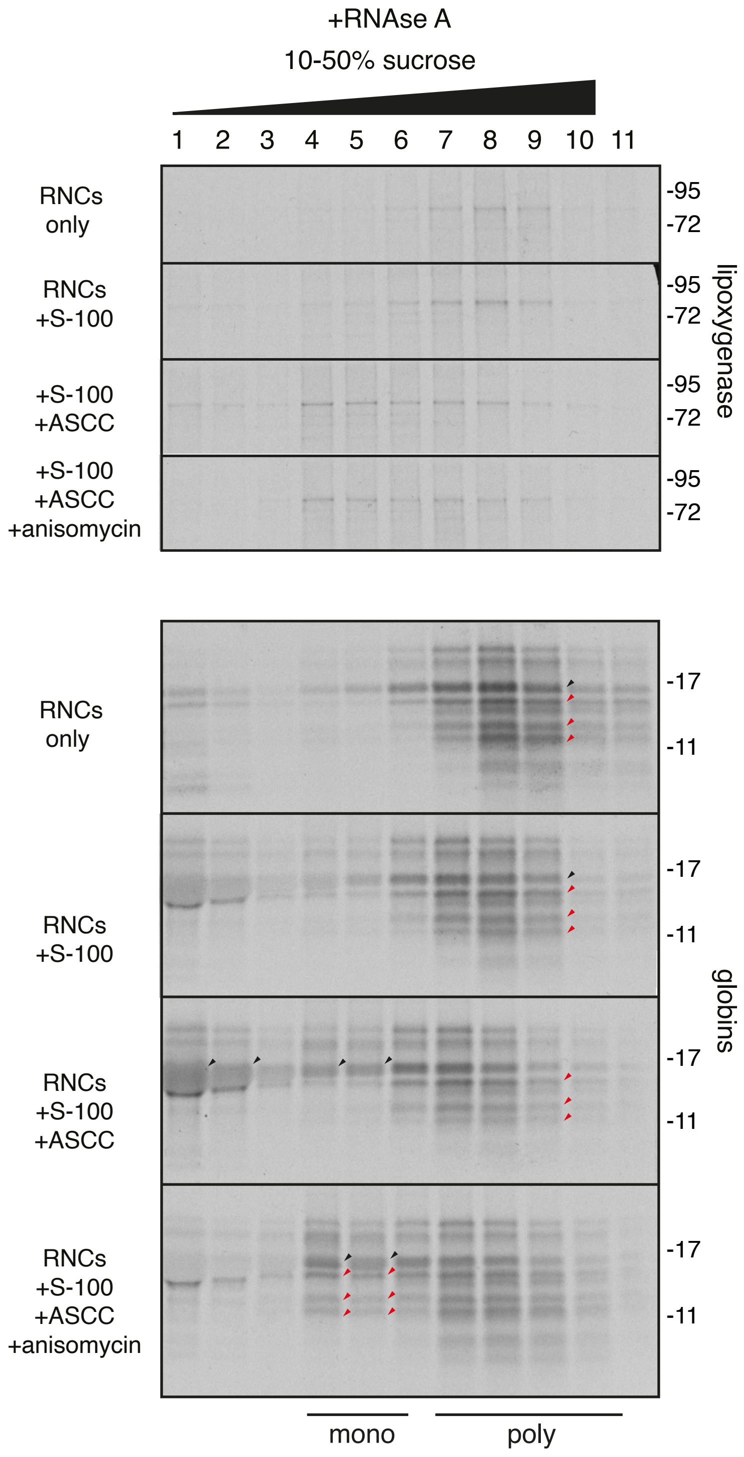

Figure S3. The lead ribosome of a queue is targeted by ASCC. Related to Figure 5.
Purified collided, ubiquitinated and radiolabelled ribosome-nascent chain complexes (RNCs) were mixed without or with S-100 obtained from RRL, ASCC, and anisomycin. After a 30 min disassembly reaction, the products of the reaction were separated on the sucrose gradients. The tRNA was digested with RNase A prior to gel electrophoresis. Top panel shows that region of the gel containing nascent lipoxygenase, whereas bottom panel shows the region of the gel containing nascent globins. Full length globins are marked with black arrows and truncated globins are marked with red arrows. Note that S-100 does not contain appreciable disassembly activity as indicated by the near-identical profiles of the "RNCs only" and "+S-100" samples. Addition of ASCC results in diassembly as indicated by the appearance of full length nascent chains in the monosome/subunit fractions (4-6). Truncated nascent chains from the trailing ribosomes are not found in these fractions unless elongation is inhibited by anisomysin during the disassembly reaction.

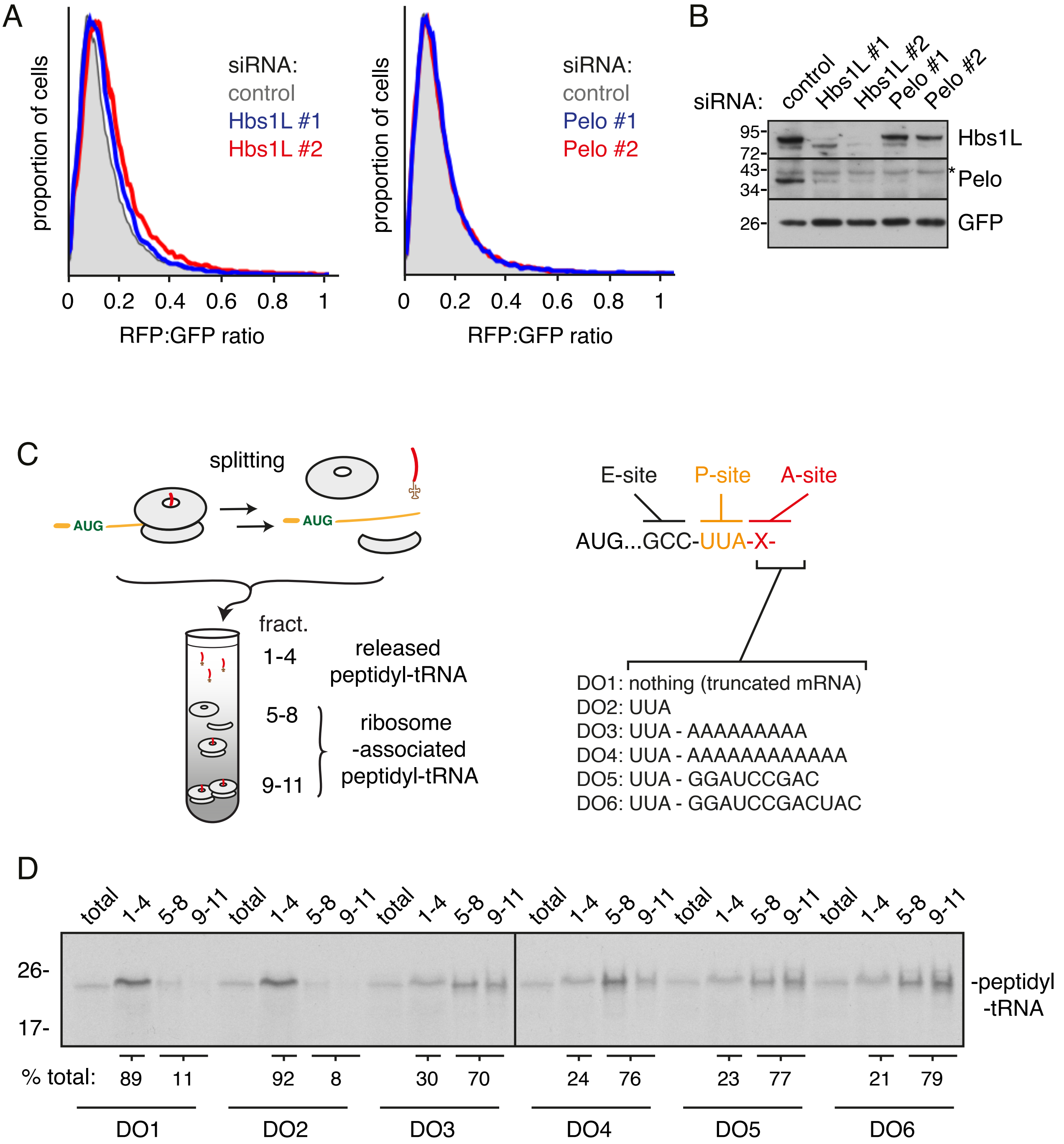

A
proportion of cells
siRNA:
control
Hbs1L #1
Hbs1L #2
0
0.2
0.4
0.6
0.8
1
RFP:GFP ratio
proportion of cells
siRNA:
control
Pelo #1
Pelo #2
RFP:GFP ratio
B
siRNA:
control
Hbs1L #1
Hbs1L #2
Pelo #1
Pelo #2
95-
72-
43-
34-
26-
Hbs1L
*Pelo
GFP
C
splitting
AUG
AUG
fract.
1-4
released peptidyl-tRNA
5-8
9-11
ribosome -associated peptidyl-tRNA
E-site
P-site
A-site
AUG...GCC-UUA-X-
DO1: nothing (truncated mRNA)
DO2: UUA
DO3: UUA - AAAAAAAAA
DO4: UUA - AAAAAAAAAAAA
DO5: UUA - GGAUCCGAC
DO6: UUA - GGAUCCGACUAC
D
total
1-4
5-8
9-11
26-
17-
-peptidyl -tRNA
% total:
89
11
92
8
30
70
24
76
23
77
21
79
DO1
DO2
DO3
DO4
DO5
DO6

Figure S4. Targets for ribosome splitting in RRL. Related to Figure 6.
(A) WT Cells with stably integrated $(K^{AAA})_{21}$ reporter (see Figure 1A) were treated with siRNAs targeting Hbs1L (left) or Pelo (right) for 72h. The fluorescent reporter was induced with doxycycline for 20 h and the cells analyzed by flow cytometry. The histogram from ~ 20,000 GFP postive cells is plotted. **(B)** Cells from panel A were analsyed by western blotting with indicated antibodies. Note that Pelo was unstable in the absence of Hbs1L. Asterisk indicates non-specific band recognized by anti-Pelo antibody.
(C) Logic of the ribosome splitting assay. An mRNA lacking a stop codon and encoding a short (<40 amino acid) protein is translated in RRL. If the stalled ribosome is split, the peptidyl-tRNA slips out of the 60S subunit and migrates near the top of a sucrose gradient. The 3' ends of the various drop-off test constructs (DO1-DO6) are shown. RRL is naturally low in the tRNA that decodes the UUA codon such that one UUA can be decoded but two sequential UUA codons result in stalling (with the second UUA in the A site). Hence, DO1 will contain an empty A site, DO2 through DO6 will contain UUA in the A site, and DO3 through DO6 will contain different sequences downstream of the A site within the mRNA channel. **(D)** Each of the mRNAs depicted in panel C was translated in RRL lacking exogenously added tRNAs and the products were separated on a sucrose gradient. Pooled fractions were analyzed by electrophoresis through Bis-Tris gels (to maintain peptidyl-tRNA bonds) and visualized by autoradiography. Fractions 1-4 contain the released peptidyl-tRNA product of splitting, while fractions 5-11 contain ribosome-associated peptidyl-tRNA. Note that these gradients were centrifuged for 30 min (unlike 20 min in other experiments shown throughout the paper) to achieve better separation of released and ribosome-associated peptidyl-tRNA species. Under these conditions, monosomes and polysomes do not segregate neatly to fractions 5-8 and 9-11, respectively. Thus, the sum of the signal in these fractions was defined as the 'ribosome-associated' population of peptidyl-tRNA. The autoradiogram was quantified and the proportion of total signal corresponding to free versus ribosome-associated peptidyl-tRNA is shown below the respective lanes. Ribosomes containing an empty A site (DO1) or with only 3 downstream nucleotides (DO2) are split efficiently, but others are not. RRL does not have detectable amounts of either ZNF598 or ASCC. Earlier studies (Pisareva et al., 2011; Shao et al., 2013) have shown that the splitting that is seen in RRL is mediatd by endogenous Pelo and Hbs1L. Furthermore, this splitting is not dependent on ribosome collisions because exogenous mRNA rarely undergoes multiple rounds of initiation in our RRL system, with ~85-90% of translation products being in monosomes (Juszkiewicz et al., 2018). Given the high amount of splitting that is seen, we can therefore deduce that collisions are not needed for Pelo/Hbs1L-mediated splitting and that the Pelo/Hbs1L system is not sufficient to disassemble collided ribosomes (e.g., Fig. 3).

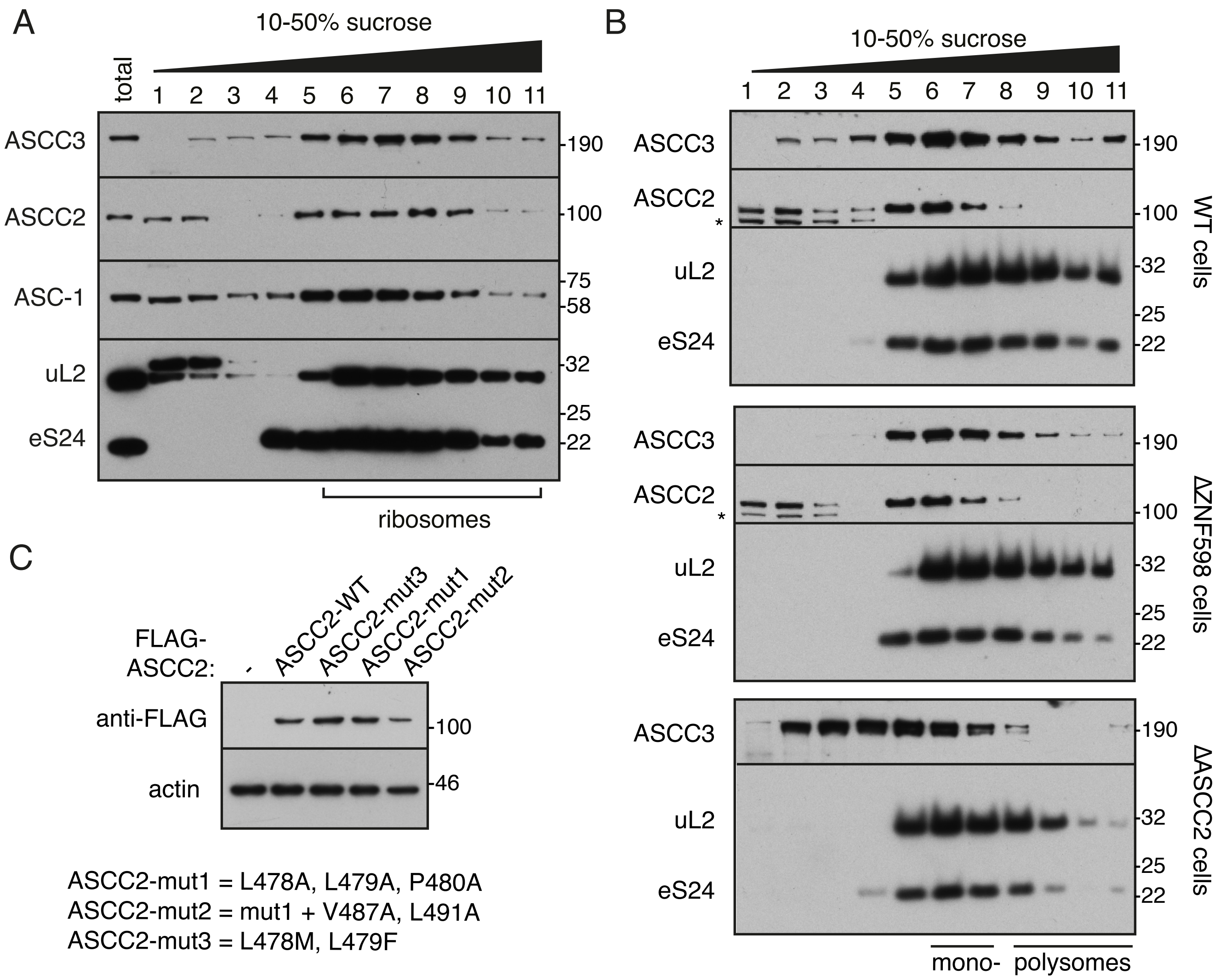

Figure S5. ASCC interacts with the ribosome independent of ZNF598. Related to Figures 1, 2, and 7. **(A)** Cytosol from HEK293 cells was fractionated on a 10-50% sucrose gradient and immunoblotted for the indicated proteins. The ASCC subunits co-fractionate predominantly with ribosomes. Antibodies against ASCC1 were insufficiently reliable to properly monitor its fractionation properties. **(B)** Sucrose gradient analysis as in panel A of cytosol extracted from the indicated cell lines. Note that ASCC2 and ASCC3 are unchanged in their fractionation properties in the absence of ZNF598. ASCC3 co-fractionation with the ribosome is impaired (but not entirely lost) in ΔASCC2 cells, and very little co-fractionation with polysomes is evident. Asterisk denotes non-specific band detected with anti-ASCC2 antibody. **(C)** Expression levels of wild type and three ASCC2 CUE-domain mutants (mut1, mut2, and mut3) in HEK293 cells upon transient transfection. ASCC2-mut1 completely abolishes the interaction with ubiquitin, ASCC2-mut2 add two additional mutations to those in mut1, and ASCC2-mut3 somewhat increases its affinity toward ubiquitin. ASCC2-mut2 is consistently expressed at lower levels than the other constructs, possibly explaining why this construct was partially impaired in its function in a recent study (Hashimoto et al., 2020). ASCC2-mut1 was the construct used in Fig. 2A.
